# CBP/p300 lysine acetyltransferases inhibit HIV-1 expression in latently infected T cells

**DOI:** 10.1016/j.isci.2024.111244

**Published:** 2024-10-28

**Authors:** Riley M. Horvath, Ivan Sadowski

**Affiliations:** 1 Department of Biochemistry and Molecular Biology, Molecular Epigenetics Group, Life Sciences Institute, University of British Columbia, Vancouver, BC, Canada

**Keywords:** Biological sciences, Immunology

## Abstract

HIV-1 latency is regulated by chromatin modifying enzymes, and histone deacetylase inhibitors (HDACi) cause reactivation of provirus expression. Surprisingly, we observed that inhibitors of the CBP/p300 acetyltransferases also cause reversal of latency in T cells. CBP/p300 inhibitors synergize with various latency reversing agents to cause HIV-1 reactivation. In contrast, inhibition of CBP/p300 impaired reversal of latency by the HDACi SAHA, indicating that CBP/p300 must contribute to acetylation on the HIV-1 LTR associated with HDACi-mediated latency reversal. CBP/p300 inhibition caused loss of H3K27ac and H3K4me3 from the LTR, but did not affect association of the inhibitor protein BRD4. Furthermore, inhibition of the additional lysine acetyltransferases PCAF/GCN5 or KAT6A/KAT6B also caused reversal of latency, suggesting that protein acetylation has an inhibitory effect on HIV-1 expression. Collectively, these observations indicate that transcription from the HIV-1 LTR is controlled both positively and negatively by protein acetylation, likely including both histone and non-histone regulatory targets.

## Highlights

• CBP/p300 inhibitors induce HIV-1 in T cell lines and CD4^+^ cells from people living with HIV
• Inhibition of CBP/p300 synergistically suppresses HIV-1 in combination with multiple LRAs
• CBP/p300 inhibitors erase H3K27ac and H3K4me3 from the LTR while causing proviral reactivation

## Graphical abstract

**Figure:**
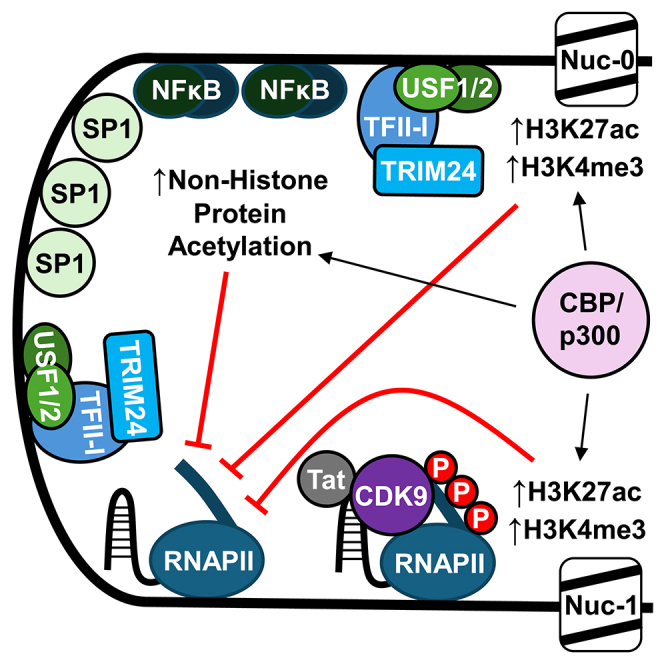

## Introduction

The advent of combination antiretroviral therapy (cART) has greatly improved the prognostic outcome of human immunodeficiency virus type 1 (HIV-1) infection by effectively managing the retrovirus as a chronic illness. However, cART does not represent a cure and must be administered lifelong as viral rebound promptly ensues following disruption of treatment in people living with HIV-1 (PLWH).^1^ The predominant barrier to a cure for HIV-1 is the extremely long-lived CD4^+^ T cells that possess chromosomally integrated but transcriptionally silent latent provirus that develop over the course of infection. This latent population serves as a reservoir of viremia that escapes immune system surveillance while remaining capable of reactivation leading to recurrent seeding of the HIV-1 infection.^2^ HIV-1 infection persists in PLWH because of latently infected cells, and consequently much attention has been placed on manipulating proviral expression with the objective of eradicating these cells or blocking stochastic reactivation in order to eliminate the requirement of continuous cART administration. Two seemingly antithetical approaches have been proposed toward potential cures, designated the “shock and kill” and the “block and lock” strategies. The proposed “shock and kill” tactic would involve application of latency reversing agents (LRAs) to induce proviral expression enabling removal of infected cells through a combination of immune system clearance and viral-mediated cytotoxicity.^3^ On the other hand, the “block and lock” approach would involve intervention with latency promoting agents (LPAs) to enforce deep latency whereby spurious proviral reactivation is inhibited in the absence of ongoing treatment.^4^^,^^5^

The “shock and kill” strategy has been intensely investigated and has led to identification of diverse LRAs through both mechanism-based approaches and small molecule compound screens.^6^^,^^7^ Multiple classes of LRAs have been described that include T cell signaling agonists,^8^ kinase agonists,^9^^,^^10^ epigenetic modifiers,^11^^,^^12^^,^^13^ transcriptional elongation enhancers,^14^ bromodomain and extra-terminal domain (BET) inhibitors,^15^^,^^16^ and TRIM24 bromodomain inhibitors.^17^^,^^18^ Several barriers to the “shock and kill” strategy have become apparent, including the need to penetrate diverse tissue types that harbor HIV-1 infected cells, including anatomical sanctuary sites, the inability to provoke a sufficiently large proviral reactivation response,^19^ and inefficient elimination of infected cells by CD8^+^ killer T cells.^20^^,^^21^ In general, a greater understanding of the mechanisms behind HIV-1 latency is required to produce a successful HIV-1 elimination strategy using this approach.

Given the obstacles to “shock and kill”, an alternative “block and lock” strategy has garnered recent attention, where the intention is to use LPAs to enforce production of deep latency where provirus expression remains suppressed in the absence of cART.^22^ One well characterized LPA is didehydro-cortistatin A (dCa), an analog of a natural steroidal alkaloid from the marine sponge *Corticium simplex*.^23^ dCA was found to inhibit the viral transactivator Tat through direct interaction, resulting in formation of a restrictive LTR epigenetic composition that limits RNAPII recruitment.^24^^,^^25^ Importantly, HIV-1 suppression is maintained following the discontinuation of dCA treatment.^26^^,^^27^ Similar to the effect of dCA, the Nullbasic Tat mutant disrupts the Tat/P-TEFb positive feedback loop resulting in suppression of proviral transcription.^28^ Additionally, inhibitors of the transcriptional mediator kinase, CDK8/CDK19, were shown to inhibit HIV-1 reactivation from latency.^29^ Furthermore, the CDK8/19 inhibitors Senexin A and BRD6989 suppressed stochastic proviral expression, an effect that is maintained following discontinuation of CDK8/19 inhibition.^29^ Although additional LPAs continue to be discovered, clinical trials have yet to be initiated for this potential therapeutic strategy.

The silencing of HIV-1 expression occurs to a significant extent via epigenetic mechanisms.^30^ HIV-1 predominately integrates into actively transcribed chromatin and becomes subject to regulation by histone modifying enzymes that promote an active or restrictive epigenetic environment on the LTR promoter.^31^ Generally, histone demethylases (HDMs) compete with the activity of histone methyltransferases (HMTs) for methylation of histone N-terminal tail lysine residues, while histone acetyltransferases (HATs) antagonize the activity of histone deacetylases (HDACs). Histone methylation is known to promote or restrict transcription dependent on the target lysine residue. For instance, H3K27me is associated with transcriptional repression while H3K4me correlates with activation. Methylation of H3K9, H3K27, and H4K20 has been shown to be associated with proviral latency where inhibition of the catalyzing HMTs causes latency reversal.^12^^,^^32^^,^^33^^,^^34^

Additionally, transcription from the HIV-1 provirus is repressed by multiple HDACs, recruited by factors, including YY1, NF-κB p50, and CBF-1,^35^ which remove acetylation from LTR-associated histones. Notably, HDAC inhibitors (HDACi) are a predominant LRA class that have already been evaluated in clinical trials,^36^^,^^37^ but to date have not reduced the size of the latent reservoir.^38^ Opposing HDAC activity are the histone/lysine acetyl transferases (HATs/KATs), including CBP/p300, which are recruited to the HIV-1 LTR upon transcriptional induction, and whose presence correlates with enrichment of histone acetylation.^39^ In particular, NFAT and Tat have been shown to facilitate the recruitment of CBP and its paralog p300 to the HIV-1 LTR.^39^ However, evidence of a direct role of CBP/p300 for the regulation of HIV-1 expression by specific acetylation of target protein(s) is lacking.

Given the association of histone acetylation and activation of HIV-1 provirus, we examined whether inhibition of CBP/p300 acetyltransferase activity would inhibit provirus reactivation, using the recently developed small molecule inhibitors A-485^40^ and iP300w.^41^ To our surprise, inhibition of CBP/p300 activity with these compounds induced HIV-1 transcription in both cell line models of latency and primary CD4^+^ peripheral blood mononuclear cells (PBMCs) *ex vivo*. When used in combination with either the MAPK/PKC agonist PMA, the NF-κB activator PEP005, the BET bromodomain inhibitor JQ1, or the TRIM24 bromodomain inhibitor IACS-9571, CBP/p300 inhibition caused synergistic and robust induction of proviral expression. Consistent with the role of CBP/p300 for acetylation dependent gene activation, both A-485 and iP300w antagonized latency reversal in response to the HDACi SAHA. Furthermore, proteolysis targeting chimera (PROTAC) mediated degradation of CBP/p300 using dCBP-1^42^ mirrored effects of the chemical inhibitors. In addition, although CBP/p300 inhibition induced HIV-1 expression, we observed the loss of H3K27ac and H3K4me3 epigenetic marks at the proviral LTR, which are generally associated with activation. This work indicates that acetylation of protein(s) by CBP/p300 causes an inhibitory effect on the HIV-1 LTR, opposite that of known effects of histone acetylation for regulation of transcription. This effect of HAT/KAT inhibitors requires consideration for development of therapeutic strategies to modulate HIV-1 expression, particularly involving HDAC inhibitors.

## Results

### CBP/p300 acetyltransferase inhibitors induce HIV-1 transcription

**Figure 1:**
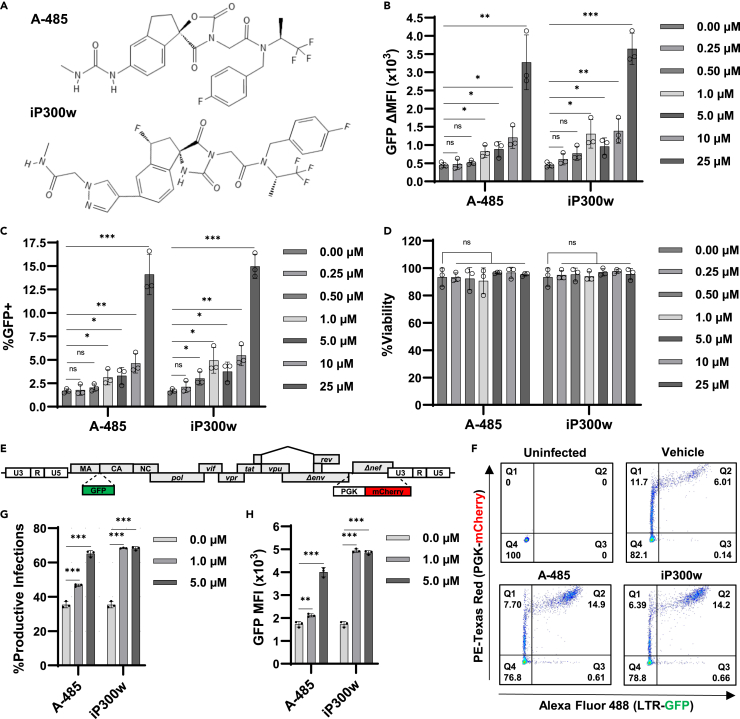
Inhibitors of CBP/p300 acetyltransferase activity promote HIV-1 transcription (A) Molecular structure of the highly selective CBP/p300 acetyltransferase inhibitors A-485 and iP300w. (B and C) JLat10.6 cells were incubated with the indicated concentration of CBP/p300 inhibitor for 24 h. Subsequently, HIV-1 expression was assessed by flow cytometry and is reported as the GFP delta (Δ) Mean Fluorescence Intensity (MFI) (B) and the proportion of GFP expressing cells (C) (*n* = 3, mean ± SD, unpaired t test). (D) Cellular viability was determined for JLat10.6 cells treated as in (B) and (C) (*n* = 3, mean ± SD, unpaired t test). (E) Schematic representation of the Red-Green-HIV-1 full-length dual reporter virus.^22^ GFP expression is directed by the 5′ LTR and is a measure of proviral transcription, while an internal constitutive PGK promoter drives mCherry expression allowing for the determination of infection independent of LTR activity. A frameshift mutation in *env* renders the virus replication incompetent. (F) Representative flow cytometry scatterplots of RGH infected Jurkat E6-1 T cells that 1-day post-infection, were treated with a vehicle control (DMSO), 5 μM A-485, or 5 μM iP300w for 48 h. Latently infected cells are in Q1 (mCherry+), productive HIV-1 infected T cells are in Q2 (GFP+/mCherry+), noise generated by viral recombination is in Q3 (GFP+), and uninfected cells are in Q4. (G and H) Jurkat E6-1 T cells were transduced with RGH at a multiplicity of infection that caused ∼20% of cells to be infected. 24 h post-infection, cells were incubated with a vehicle control (DMSO), or the indicated concentration of A-485 or iP300w. Following 48 h, HIV-1 expression was determined by flow cytometry and is reported as the percent of productively infected cells (Q2/(Q1+Q2)x100) (G) and the GFP Mean Fluorescence Intensity (MFI) of the mCherry+ population (Q1 and Q2) (H) (*n* = 3, mean ± SD, unpaired t test). Statistical significance is indicated at ∗*p* < 0.05, ∗∗*p* < 0.01, or ∗∗∗*p* < 0.001, with n.s. denoting non-significant *p* ≥ 0.05.

Given the presumption that CBP/p300 acetyltransferases are associated with reactivation of proviral latency,^30^^,^^43^ we sought to characterize the effect of CBP/p300 chemical inhibitors on HIV-1 transcription. To this end, we treated the JLat10.6 T cell model of HIV-1 latency with A-485^40^ and iP300w,^41^ two recently developed small molecules that are highly specific inhibitors of CBP/p300 acetyltransferase activity (Figure 1A). The JLat10.6 cell line possesses a chromosomally integrated provirus where GFP is inserted in place of *nef* and serves as a readout for HIV-1 5′ LTR expression, while a frameshift in *env* prevents additional rounds of replication.^44^ To our surprise, we observed that the inhibitors caused a dose-dependent increase in HIV-1 expression as measured by 5′ LTR driven GFP expression (Figures 1B and 1C). Treatment of cells with A-485 and iP300w for 24 h produced a modest 2.7- and 3.1-fold induction in HIV-1 expression, respectively (Figure 1 and 24 h), but expression increased considerably after two days treatment to 6.5x for A-485 and 8.9x for iP300w (Figure 1 and 48 h). Importantly, the inhibitors did not generate autofluorescence (Figure S2) and cell viability was unaffected at the concentrations used (Figure 1D).

Since the establishment of latency can occur early upon infection,^22^^,^^31^^,^^45^ we examined the effect of CBP/p300 inhibition following infection of T cells with the Red-Green-HIV-1 (RGH) dual fluorescence reporter virus.^22^ RGH is a full-length HIV-1 reporter where GFP is expressed from the 5′ LTR, and a constitutive promoter expressing mCherry is inserted in place of *nef* to allow differentiation between latent and productively infected cells (mCherry+/GFP−, mCherry+/GFP+, respectively). Additionally, a frameshift mutation in *env* renders this virus replication incompetent, preventing multiple rounds of infection (Figure 1E). Following infection of Jurkat CD4^+^ T cells with RGH, treatment with CBP/p300 inhibitors caused an increase in the proportion of productive infections (Figures 1F and 1G) and resulted in a significant increase in expression from the 5′ LTR (Figure 1H). Collectively, these results suggest that CBP/p300 acetyltransferase activity causes repression of HIV-1 expression and contributes to the development of latency upon infection.

### CBP/p300 inhibitors synergize with mechanistically distinct LRAs for HIV-1 reactivation

**Figure 2:**
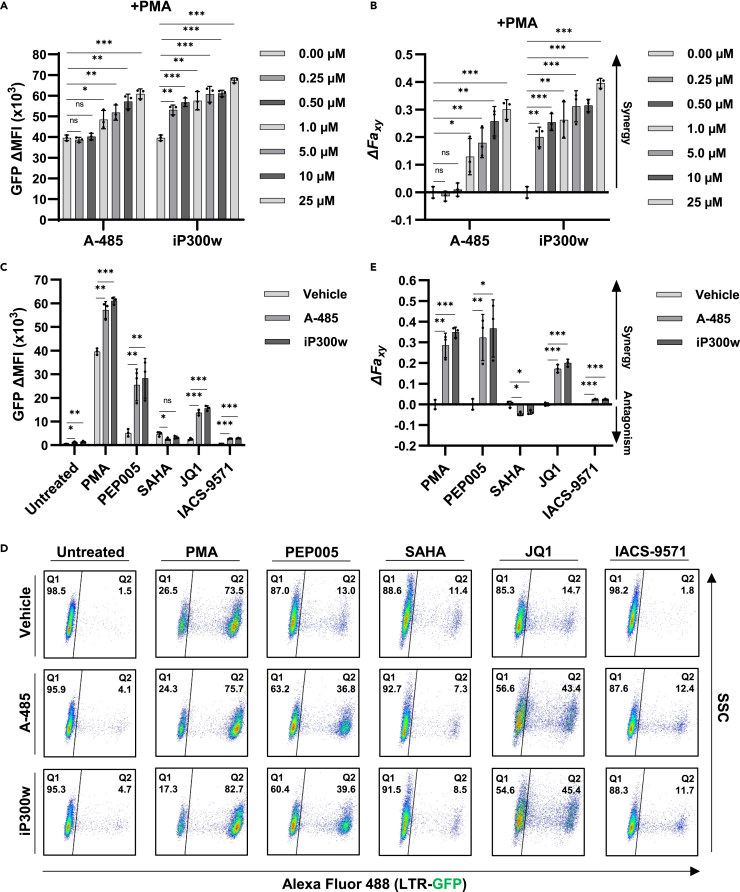
Effect of CBP/p300 inhibitors in combination with LRAs (A) JLat10.6 cells were pre-treated with the indicated concentration of A-485 or iP300w for 1 h, after which 4 nM PMA was added. Following 24 h, HIV-1 expression was determined by flow cytometry and is indicated as the GFP delta (Δ) mean fluorescence intensity (MFI) (*n* = 3, mean ± SD, unpaired t test). (B) Drug interaction between 4 nM PMA and the indicated concentration of either A-485 or iP300w was determined using Bliss Independence Modeling. Presented is the difference between the predicted and the observed fractional HIV-1 transcription response to the given drug combination whereby values greater than 0 indicate a synergistic interaction. See Materials and Methods for more details (*n* = 3, mean ± SD, unpaired t test). (C) JLat10.6 cells were treated with a vehicle control (DMSO), 10 μM A-485, or 10 μM iP300w for 1 h prior to the addition of the indicated latency reversing agent. Following 24 h, HIV-1 expression was assessed by flow cytometry and is reported as the GFP delta (Δ) mean fluorescence intensity (MFI). The concentration of LRAs used were 4 nM PMA, 4 nM PEP005, 1 μM SAHA, 10 μM JQ1, 15 μM IACS-9571 (*n* = 3, mean ± SD, unpaired t test). (D) Representative flow cytometry scatterplots for JLat10.6 cells treated as indicated in (C). Q1 contains latent (GFP-) cells while cells possessing transcriptionally active provirus are shown in Q2 (GFP+). (E) Bliss Independence Modeling was applied to determine combinatorial drug interaction as in (B). Values greater than 0 indicate synergy while values less than 0 signify an antagonistic relationship (*n* = 3, mean ± SD, unpaired t test). Statistical significance is indicated at ∗*p* < 0.05, ∗∗*p* < 0.01, or ∗∗∗*p* < 0.001, with n.s. denoting non-significant *p* ≥ 0.05.

As CBP/p300 inhibition was found to reverse HIV-1 latency, we examined the effect of A-485 and iP300w in combination with various mechanistically distinct LRAs. The phorbol ester PMA is a Ras-MAPK/PKC agonist that reactivates latent HIV-1 by stimulating factors including NF-κB, AP1, and GABP/ETS.^46^ Treatment of JLat10.6 T cells with PMA in combination with a range of CBP/p300 inhibitor concentrations revealed robust and synergistic activation of HIV-1 expression as determined by Bliss Independence Modeling (Figures 2A and 2B). Of note, both inhibitors generated a dose-dependent increase in HIV-1 expression that was greater than treatment with PMA alone (Figure 2A), although lower concentrations of iP300w than A-485 were required to produce synergistic activation (Figure 2B). Additionally, no effect on cellular viability was observed for any of the treatments (Figure S3A).

Next, we compared the effect of CBP/p300 inhibitors in combination with the additional previously characterized LRAs PEP005, SAHA, JQ1, and IACS-9571. PEP005 causes PKC dependent NF-κB activation; SAHA is a pan-HDACi; JQ1 is a BET protein (BRD4) bromodomain inhibitor; and IACS-9571 is a TRIM24 bromodomain inhibitor that stimulates HIV-1 transcriptional elongation.^17^^,^^18^ We observed significant reversal of proviral latency for each combination of CBP/p300 inhibitor and LRA as compared to individual treatment, with the exception of SAHA where the combination with CBP/p300 inhibitor slightly inhibited transcriptional induction (Figures 2C and 2D). Bliss Independence Modeling confirmed that CBP/p300 inhibitors cause a synergistic effect with PMA, PEP005, JQ1, and IACS-9571, but are antagonistic toward SAHA (Figure 2E). Importantly, cellular toxicity was not observed for any combination of drugs examined (Figure S3B). Because SAHA is an HDACi that reverses latency, presumably by enhancing LTR associated histone acetylation, the finding that CBP/p300 inhibitors reduce SAHA mediated HIV-1 induction indicates that CBP/p300 must contribute to accumulation of protein acetylation associated with activation of transcription from the HIV-1 LTR. However, we note that multiple HATs must be required for the latency reversing effect of SAHA because CBP/p300 inhibitors only partially prevent reactivation. More importantly, because inhibition of CBP/p300 reverses proviral latency (Figure 1) and synergizes with other LRAs for reactivation of HIV-1 provirus, additional uncharacterized protein acetylation must cause repression of HIV-1 transcription.

### PROTAC mediated CBP/p300 degradation reactivates latent HIV-1

**Figure 3:**
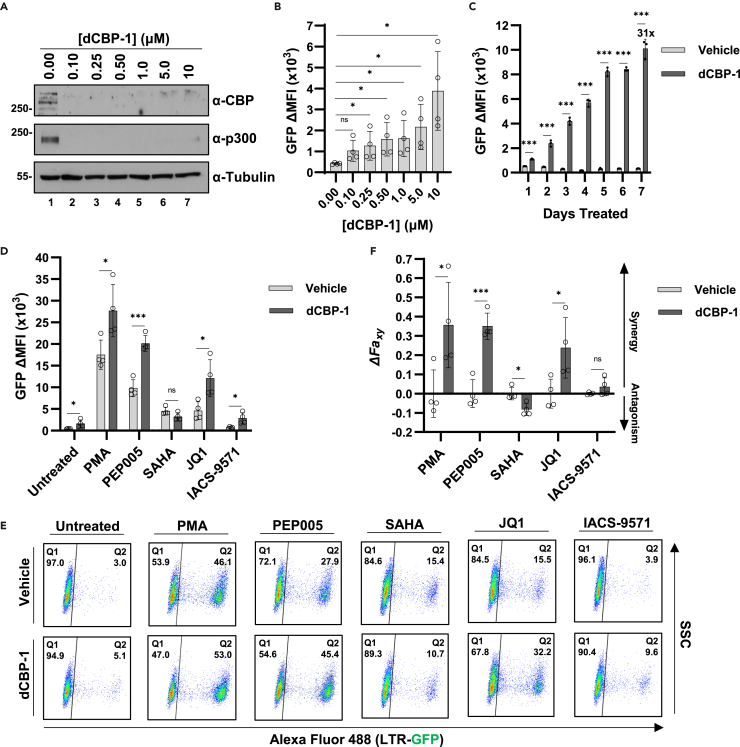
PROTAC-mediated degradation of CBP/p300 reactivates latent HIV-1 (A) JLat10.6 cells were incubated with the indicated concentration of dCBP-1. Following 24 h, whole cell lysates were extracted and subjected to immunoblotting with the indicated antibody. (B) JLat10.6 cells were incubated for 24 h with the indicated concentration of dCBP-1. After 24 h, HIV-1 expression was assessed by flow cytometry and is depicted as the change (Δ) in GFP mean fluorescence intensity (MFI) (*n* = 4, mean ± SD, unpaired t test). (C) JLat10.6 cells were incubated with a vehicle control or 1 μM dCBP-1. Following the indicated amount of time, HIV-1 expression was assessed by flow cytometry and is reported as the GFP delta (Δ) mean fluorescence intensity (MFI) (*n* = 3, mean ± SD, unpaired t test). (D) JLat10.6 cells were incubated in the presence of 1 μM dCBP-1 for 3 h. Subsequently, the indicated LRA was added, and HIV-1 expression was examined 24 h later by flow cytometry and is depicted as the delta (Δ) GFP mean fluorescence intensity (MFI). The concentrations of LRAs used were 4 nM PMA, 4 nM PEP005, 1 μM SAHA, 10 μM JQ1, 15 μM IACS-9571 (*n* = 4, mean ± SD, unpaired t test). (E) Representative flow cytometry scatterplots for JLat10.6 cells treated as indicated in (D). Q1 contains latent (GFP−) cells while the transcriptionally active population is present in Q2 (GFP+). (F) Bliss Independence Modeling was applied to determine combinatorial drug interactions between dCBP-1 and the indicated LRA. Values greater than 0 indicate synergy while values less than 0 signify an antagonistic relationship (*n* = 4, mean ± SD, unpaired t test). Statistical significance is indicated at ∗*p* < 0.05, ∗∗*p* < 0.01, or ∗∗∗*p* < 0.001, with n.s. denoting non-significant *p* ≥ 0.05.

Given the surprising finding that inhibitors of CBP/p300 acetyltransferase activity cause reactivation of HIV-1 expression, we sought to confirm this effect using dCBP-1, a PROTAC consisting of a CBP/p300 bromodomain binding motif and the cereblon E3 ubiquitin ligase targeting moiety that causes the proteasomal degradation of CBP and p300.^42^ We first confirmed a range of dCBP-1 concentrations that causes the near total degradation of CBP and p300 proteins within 24 h (Figure 3A). This range is consistent with previous findings where higher concentrations were found to trigger more rapid proteolysis.^42^ Treatment of JLat10.6 T cells with dCBP-1 caused dose-dependent induction of HIV-1 expression (Figure 3B), the magnitude of which mirrored CBP/p300 acetyltransferase inhibition (Figure 1B). As with the acetyltransferase inhibitors (Figure S1), treatment with dCBP-1 over a week resulted in a gradual increase in HIV-1 expression, peaking at 31-fold greater than untreated cells (Figure 3C). Next, we applied the CBP/p300 PROTAC in combination with LRAs to examine synergistic effects for reactivation of HIV-1 provirus. Similar to the previous results with the CBP/p300 acetyltransferase inhibitors, dCBP-1 in combination with PMA, PEP005, JQ1, or IACS-9571 generated greater HIV-1 expression than individual treatment, while causing a slight decrease in SAHA-mediated latency reversal (Figures 3D and 3E). As with A-485 or iP300w, we observed synergistic effects of dCBP-1 with PMA, PEP005, JQ1, and IACS-9571, and an antagonistic effect with SAHA (Figure 3F). Treatment with dCBP-1 alone or in combination with the LRAs had no effect on cellular viability (Figure S4). Taken together, these results confirm a repressive function of CBP/p300 on HIV-1 expression.

### CBP/p300 inhibition induces HIV-1 expression *ex vivo*

**Figure 4:**
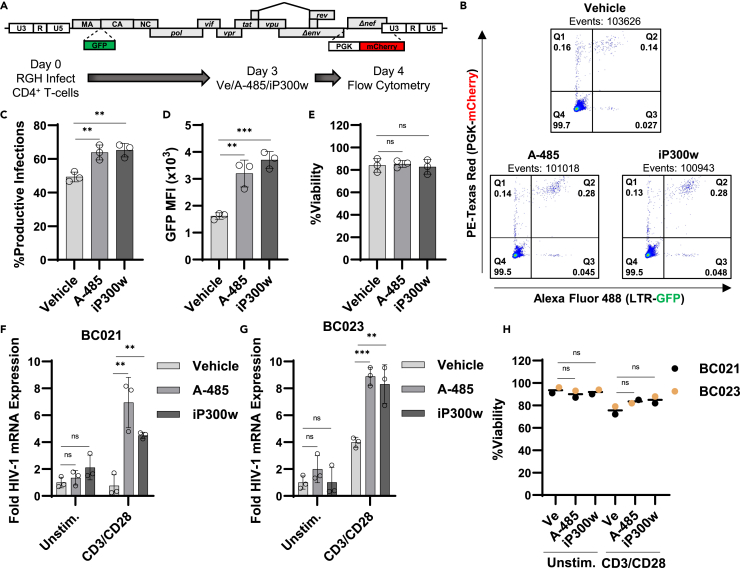
CBP/p300 inhibition induces HIV-1 expression in CD4^+^ cells *ex vivo* (A) Experimental design for RGH infection of primary CD4^+^ T cells. CD4^+^ T cells isolated from an uninfected participant were infected with the RGH dual reporter virus at a low MOI. 3 days, post-infection, cells were incubated with a vehicle control (DMSO), 10 μM A-485, or 10 μM iP300w for 24 h after which proviral expression was assessed by flow cytometry. (B) Representative flow cytometry scatterplots of primary CD4^+^ T cells treated as in (A). Latent infections are in Q1 (mCherry+), productively infected T cells are in Q2 (GFP+/mCherry+), noise generated by viral recombination is in Q3 (GFP+), and uninfected cells are in Q4. (C and D) Following treatment of primary CD4^+^ T cells as in (A), proviral transcription was determined by flow cytometry and is expressed as the percentage of productive infections (C) and the GFP mean fluorescence intensity (MFI) of infected cells (D) (*n* = 3, mean ± SD, one-way ANOVA with Dunnett’s multiple comparisons test). (E) Viability was determined for participant derived CD4^+^ T cells treated as in (A) (*n* = 3, mean ± SD, one-way ANOVA with Dunnett’s multiple comparisons test). (F and G) Primary CD4^+^ PBMCs isolated from people living with HIV-1 who are receiving ART were treated with a vehicle (DMSO), 10 μM A-485, or 10 μM iP300w and were either left unstimulated or were treated with anti-CD3/anti-CD28. Following 24 h, intracellular RNA was extracted, and RT-PCR was preformed using oligos specific for multiply spliced Tat-Rev HIV-1 mRNA transcripts. HIV-1 mRNA expression is normalized to *GAPDH* (*n* = 3, mean ± SD, unpaired t test). (H) Primary CD4^+^ PBMCs treated as in (F) and (G) were assessed for cellular viability (*n* = 2, mean, unpaired t test). Statistical significance is indicated at ∗*p* < 0.05, ∗∗*p* < 0.01, or ∗∗∗*p* < 0.001, with n.s. denoting non-significant *p* ≥ 0.05.

We also assessed the effect of CBP/p300 inhibition on HIV-1 expression *ex vivo*. For this, we infected primary CD4^+^ T cells isolated from a healthy individual with the RGH dual reporter virus. 3 days post-infection, the cells were treated with A-485 or iP300w for 24 h (Figure 4A). Following treatment with CBP/p300 inhibitor, we observed a marginal increase in the proportion of productive infections (Figures 4B and 4C) and ∼2-fold increase in expression from the 5′ LTR as measured by GFP expression (Figure 4D). Treatment with the inhibitors did not have a measurable impact on cellular viability (Figure 4E).

To further evaluate the role of CBP/p300 for HIV-1 latency, we tested the propensity of CBP/p300 inhibition to reactivate HIV-1 in CD4^+^ PBMCs obtained from two aviremic participants who have been receiving cART. Treatment of cells from either participant with the CBP/p300 inhibitors on their own for 24 h did not produce a significant increase in HIV-1 mRNA (Figures 4F and 4G, Unstim.). Latent proviruses can be induced by stimuli such as cytokine or antigenic stimulation that causes cellular activation through the T cell receptor (TCR), which can be mimicked by treatment with CD3/CD28 antibodies, which cross link and activate the TCR. In CD4^+^ cells from donors stimulated by TCR cross linking we observed an increase in HIV-1 mRNA for participant BC023 but not BC021 (Figures 4F and 4G, CD3/CD28, Vehicle). However, stimulation by TCR crosslinking in combination with either A-485 or iP300w produced a significantly greater increase in HIV-1 mRNA for cells from both donors (Figures 4F and 4G). In addition, we did not observe cellular toxicity in these cells upon CBP/p300 inhibitor treatment (Figure 4H). For people living with HIV-1 (PLWH) who are receiving cART, studies indicate that roughly 1 per 10^6^ CD4^+^ T cells possess a replication competent proviral genome.^47^ That being the case, it is possible that longer treatment intervals with CBP/p300 inhibitors alone would reveal a detectable change in HIV-1 mRNA. Nevertheless, these results indicate that CBP/p300 activity contributes to repression of HIV-1 provirus in PLWH.

### CBP/p300 inhibition does not cause global T cell activation

Stimulation of the TCR through engagement with antigen-presenting cells stimulates T cell activation through the activity of TCR-associated protein tyrosine kinases which trigger the MAPK, PKC, and calcineurin pathways.^46^ These pathways activate downstream transcription factors, including NF-κB, NFAT, AP1, and GABP/Ets, which coordinate the inflammatory immune response by inducing expression of their respective target genes.^48^ Intriguingly, the HIV-1 LTR has evolved to possess binding sites for many TCR induced transcription factors, including those mentioned previously, thus coupling proviral transcription to T cell activation.^46^ Consequently, a major challenge to the “shock and kill” therapeutic strategy is to force proviral transcription without promoting global T cell activation which can cause cytokine release syndrome, a life-threatening condition.^49^

**Figure 5:**
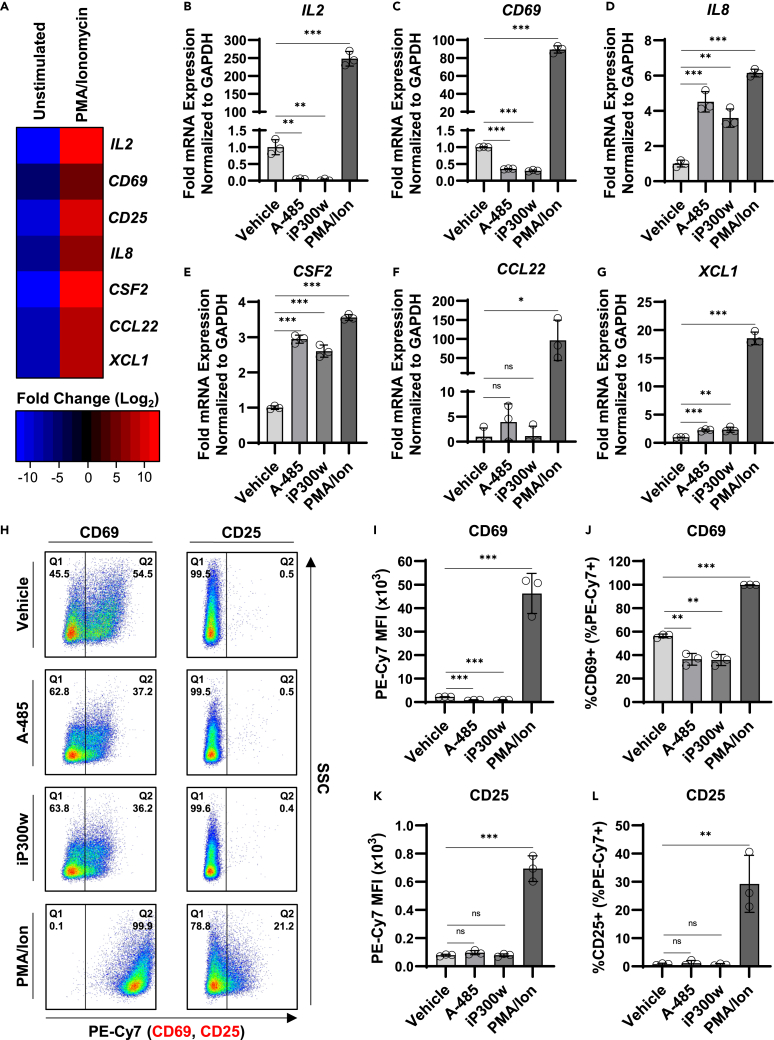
Effect of CBP/p300 inhibition on T cell activation (A) Heatmap depiction of selected upregulated genes following PMA/ionomycin treatment of Jurkat T cells as identified by RNA-seq analysis.^18^ (B–G) Jurkat T cells were incubated 24 h in the presence of DMSO (Vehicle), 10 μM A-485, 10 μM iP300w, or 4 nM PMA/1 μM ionomycin. Subsequently, intracellular RNA was extracted and analyzed by RT-PCR using oligos specific for the indicated mRNA transcript. RNA expression is normalized to *GAPDH* transcript (*n* = 3, mean ± SD, unpaired t test). (H) Representative flow cytometry scatterplots following surface staining of CD69 or CD25 receptors. Prior to membrane receptor staining, Jurkat T cells were incubated for 24 h with a vehicle control (DMSO), 10 μM A-485, 10 μM iP300w, or 4 nM PMA/1 μM ionomycin. (I and J) Following treatment of Jurkat T cells as in (H), surface staining was performed using PE-Cy7 conjugated anti-CD69. CD69 expression was determined by flow cytometric analysis and is reported as the Mean Fluorescence Intensity (MFI) of PE-Cy7 (I) and the percentage of cells presenting surface CD69 (J) (*n* = 3, mean ± SD, unpaired *t*-test). (K.and L) As in (I, J) but surface staining was performed using PE-Cy7 conjugated anti-CD25 (*n* = 3, mean ± SD, unpaired *t*-test). Statistical significance is indicated at ∗*p* < 0.05, ∗∗*p* < 0.01, or ∗∗∗*p* < 0.001, with n.s. denoting non-significant *p* ≥ 0.05.

In order to determine the effect of CBP/p300 inhibition on T cell activation, we assessed available RNA-seq data to identify genes that are differentially regulated upon PMA/ionomycin stimulation.^18^ This analysis identified 1481 downregulated genes and 2442 upregulated genes in activated CD4^+^ T cells relative to unstimulated (Figure S5). Notably, CBP (*CREBBP*) and p300 (*EP300*) expression are not altered during T cell stimulation (Figure S5). From this analysis, we selected several genes encoding cytokines and surface receptors for analysis, including *IL2* and *CD69* which are known to be upregulated upon activation^50^ (Figure 5A). Using RT-PCR, we confirmed that PMA/ionomycin mediated T cell activation induced expression of these genes (Figures 5B–5G, PMA/Ion). Interestingly, we observed that CBP/p300 inhibitors caused a variety of effects on these PMA/ionomycin-inducible genes. CBP/p300 inhibition was found to repress *IL2* and *CD69* expression, two genes that were strongly induced by T cell activation (Figures 5B and 5C). However, both A-485 and iP300w caused activation of *IL8* and *CSF2* expression, although these genes are only moderately upregulated (Figures 5D and 5E). Finally, we found that the CBP/p300 inhibitors had minimal effect on the expression of *CCL22* and *XCL1* (Figures 5F and 5G).

In addition to assessing transcript abundance, we examined surface expression of CD69 and CD25 membrane receptors. Consistent with RNA-seq analysis, PMA/ionomycin treatment resulted in enhanced surface expression of both CD69 (Figures 5H–5J, PMA/Ion) and CD25 (Figures 5H, 5K, and 5L, PMA/Ion). In contrast, CBP/p300 inhibition caused a decrease in CD69 surface expression as measured by the fluorescent intensity of stained CD69 (Figure 5I) or the proportion of cells presenting surface CD69 (Figure 5J). Depletion of surface CD69 receptor in response to CBP/p300 inhibition is consistent with the observed decrease in *CD69* mRNA transcript abundance (Figure 5C). While PMA/ionomycin treatment enhanced surface CD25 expression, no effect on expression was observed in response to CBP/p300 acetyltransferase inhibition (Figures 5K and 5L). Collectively, these results indicate that CBP/p300 inhibition does not initiate global T cell activation and likely does not promote HIV-1 latency reversal by appropriating a master immune response factor.

### Dependence of Tat for regulation of HIV-1 by CBP/p300

**Figure 6:**
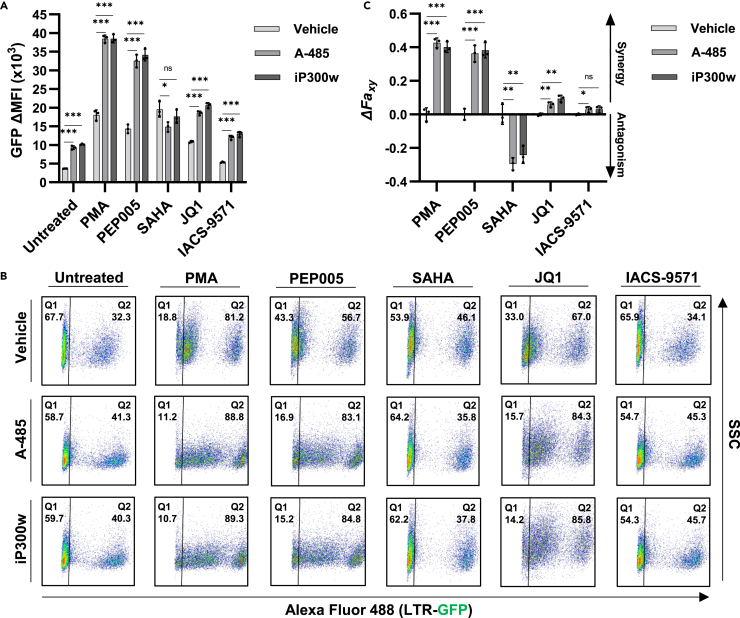
Tat dependency for CBP/p300 regulation of HIV-1 (A) JLatA72 cells possessing an integrated HIV-1 LTR-GFP reporter that does not express Tat, were incubated 1 h with a vehicle control (DMSO), 10 μM A-485, or 10 μM iP300w prior to the addition of 4 nM PMA, 4 nM PEP005, 1 μM SAHA, 10 μM JQ1, or 15 μM IACS-9571. Following 24 h, LTR transcriptional activity was assessed by flow cytometry and is reported as the GFP delta (Δ) mean fluorescence intensity (MFI) (*n* = 3, mean ± SD, unpaired t test). (B) Representative flow cytometry scatterplots of JLatA72 cells treated as in (A). Cells harboring transcriptionally silent provirus (GFP−) are located in Q1 while cells with LTR expression (GFP+) are in Q2. (C) Determination of synergy between A-485 and iP300w with the indicated latency reversing agent using Bliss Independence Modeling. Drug concentrations are the same as those used in (A). Data are presented as the difference between the predicted and the observed fractional HIV-1 expression response to the given drug combination. See Materials and Methods for more details (*n* = 3, mean ± SD, unpaired t test). Statistical significance is indicated at ∗*p* < 0.05, ∗∗*p* < 0.01, or ∗∗∗*p* < 0.001, with n.s. denoting non-significant *p* ≥ 0.05.

The viral transactivator of transcription Tat is expressed from a spliced sub-genomic HIV-1 mRNA and produces a strong positive feedback loop by recruiting *P*-TEFb, a heterodimer of CDK9 and Cyclin T1, to the 5′ LTR through interaction with the nascent TAR RNA, which facilitates escape of paused RNA polymerase II (RNAPII).^51^ Furthermore, CBP/p300 recruitment to the LTR has been shown to be facilitated by Tat, and CBP/p300 acetylation of Tat is thought to increase its transcriptional activator function.^39^^,^^52^^,^^53^^,^^54^ Given the role of Tat for HIV-1 expression and observed role of CBP/p300 for its effect, we examined the requirement of Tat for HIV-1 reactivation in response to CBP/p300 inhibition using the JLatA72 cell line. This Jurkat derived CD4^+^ T cell line harbors a chromosomally integrated HIV-1 LTR that controls GFP expression independently from Tat.^55^ Treatment of JLatA72 cells with either A-485 or iP300w caused induction of GFP expressed from the LTR, indicating that Tat is not required for stimulation of HIV-1 expression by the CBP/p300 inhibitors (Figures 6A and 6B, Untreated). Additionally, treatment with CBP/p300 inhibitors in combination with PMA, PEP005, JQ1, or IACS-9571 caused much higher levels of HIV-1 expression than any treatment alone (Figures 6A and 6B), but CBP/p300 inhibition restricted latency reversal in response to the HDACi SAHA (Figures 6A and 6B, SAHA). Bliss Independence Modeling demonstrated synergistic relationships, consistent with the previous experiments, although CBP/p300 inhibitors and JQ1 produced a smaller synergistic effect in JLatA72 cells than with the JLat10.6 cell line (Figures 2E and 6C). Collectively, these results indicate that CBP/p300 inhibits HIV-1 expression independently of Tat.

### Effect of CBP/p300 acetyltransferase inhibition on LTR histone modification

**Figure 7:**
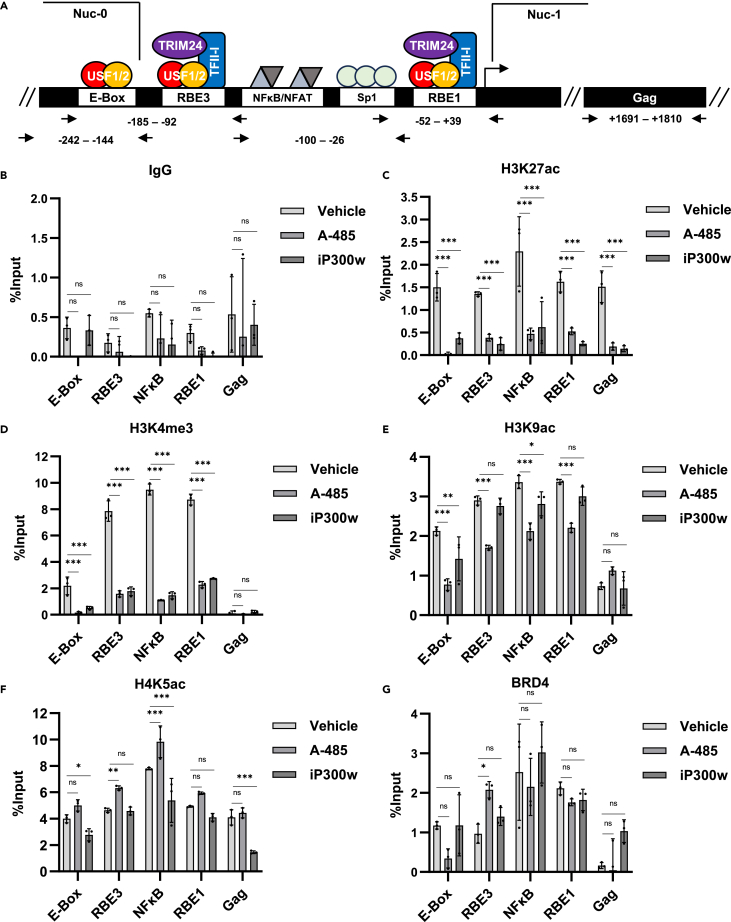
Effect of CBP/p300 inhibition on LTR epigenetic context (A) Schematic representation of the HIV-1 LTR and the primer pairs used for ChIP-qPCR analysis. (B) JLat10.6 cells were incubated with a vehicle control (DMSO), 10 μM A-485, or 10 μM iP300w for 24 h. Subsequently, ChIP was performed using non-specific rabbit IgG and the associated chromatin was analyzed by qPCR (*n* = 3, mean ± SD, two-way ANOVA with Dunnett’s multiple comparisons test). (C–G) Following treatment of JLat10.6 cells as stated in (B), ChIP was performed using antibodies specific to H3K27ac (C), H3K4me3 (D), H3K9ac (E), H4K5ac (F), or BRD4 (G). Subsequently, enrichment of chromatin was determined by qPCR using oligos directed at the indicated region (*n* = 3, mean ± SD, two-way ANOVA with Dunnett’s multiple comparisons test). Statistical significance is indicated at ∗*p* < 0.05, ∗∗*p* < 0.01, or ∗∗∗*p* < 0.001, with n.s. denoting non-significant *p* ≥ 0.05.

The previous results indicate that CBP/p300 inhibition reactivates latent HIV-1 while synergizing with various mechanistically distinct LRAs to produce robust expression. However, inhibition of CBP/p300 activity slightly impairs latency reversal in response to the HDACi SAHA (Figures 2C, 3D, and 6C). Taken together, these results indicate that CBP/p300 may modulate the LTR epigenetic environment to repress HIV-1 transcription. To examine this possibility, we performed chromatin immunoprecipitation and analysis by quantitative PCR (ChIP-qPCR) using primer sets spanning regulatory regions on the HIV-1 LTR (Figure 7A). We first performed ChIP-qPCR using non-specific IgG to establish a background signal (Figure 7B). Acetylation of H3K27 is catalyzed by CBP/p300,^56^^,^^57^^,^^58^^,^^59^ and unsurprisingly, inhibition of CBP/p300 caused drastic reduction of H3K27ac on histones associated with all regions of HIV-1 LTR (Figure 7C). Acetylation of H3K27 is typically associated with tri-methylation of H3K4 as a result of molecular crosstalk,^60^^,^^61^^,^^62^ and therefore, we examined the effect of CBP/p300 inhibitors on LTR-associated H3K4me3. Consistent with the loss of H3K27ac, CBP/p300 inhibition caused decreased H3K4me3 associated with the HIV-1 promoter (Figure 7D). We also examined the effect of CBP/p300 inhibitors on H3K9ac and H4K5ac on LTR-associated histones. Unlike H3K27ac, acetylation of H3K9 and H4K5 are only weakly associated with CBP/p300 activity.^57^ Consistently, we found that H3K9ac and H4K5ac at the LTR was largely unperturbed upon by the CBP/p300 inhibitors (Figures 7E and 7F). Recent studies indicate that histone H4 acetylation recruits BRD4 to the LTR causing reduced proviral transcription, an effect that may contribute to inhibition of HIV-1 transcription.^63^^,^^64^ However, we observed no change in association of BRD4 with the LTR in response to CBP/p300 inhibition (Figure 7G).

**Figure 8:**
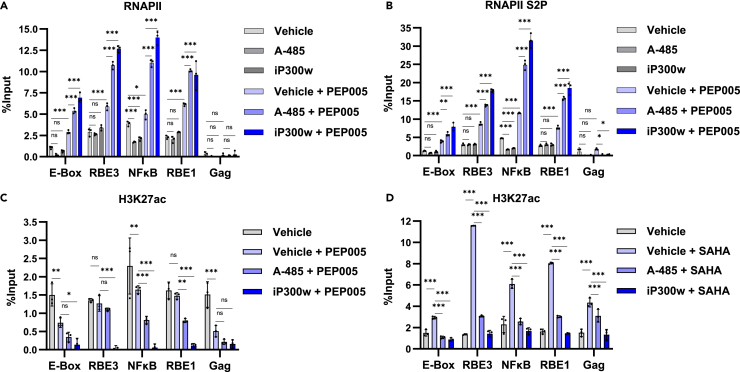
CBP/p300 inhibition primes the LTR for RNAPII recruitment (A and B) JLat10.6 cells were treated with a vehicle control (DMSO), 10 μM A-485, or 10 μM iP300w or were pre-treated 1 h with a vehicle control (DMSO), 10 μM A-485, or 10 μM iP300w prior to the addition of 4 nM PEP005. Following 24 h, ChIP was performed using antibodies specific to RNAPII (A) or S2P modified RNAPII (B), and chromatin was assessed by qPCR (*n* = 3, mean ± SD, two-way ANOVA with Dunnett’s multiple comparisons test). (C and D) JLat10.6 cells were left untreated (Vehicle, DMSO) or were pre-treated 1 h with a vehicle control (DMSO) 10 μM A-485, or 10 μM iP300w prior to the addition of 4 nM PEP005 (C) or 1 μM SAHA (D). Following 24 h, ChIP was performed using anti-H3K27ac and analyzed by qPCR (*n* = 3, mean ± SD, two-way ANOVA with Dunnett’s multiple comparisons test). Statistical significance is indicated at ∗*p* < 0.05, ∗∗*p* < 0.01, or ∗∗∗*p* < 0.001, with n.s. denoting non-significant *p* ≥ 0.05.

Aforementioned, we found that CBP/p300 inhibition results in eviction of both H3K27ac and H3K4me3 from the HIV-1 LTR (Figures 7C and 7D). Because enrichment of these epigenetic marks are typically associated with transcriptional activation, we examined the effect of CBP/p300 inhibitors on RNAPII recruitment and elongation. In this analysis, we found that treatment with CBP/p300 inhibitors in combination with the PKC-NFκB agonist PEP005, caused substantial recruitment of RNAPII and Serine 2 phosphorylated (S2P) RNAPII to the HIV-1 promoter (Figures 8A and 8B). Phosphorylation of S2 of RNAPII CTD is associated with elongation, and because enrichment of RNAPII S2P is proportional to that of RNAPII, the predominant effect of CBP/p300 inhibitors likely involves recruitment of RNAPII to the LTR promoter. Given the observation that CBP/p300 inhibition potentiates RNAPII recruitment, we assessed the effect of CBP/p300 inhibitors in combination with PEP005 or SAHA on LTR-associated H3K27ac. Unexpectedly, we observed that stimulation of PKC-NFκB by PEP005 did not cause enrichment of H3K27ac at the LTR (Figure 8C). In contrast, the HDACi SAHA caused a significant increase in H3K27ac at the viral promoter (Figure 8D). Importantly, we found that CBP/p300 inhibitors reduced H3K27ac on the LTR regardless of LRA treatment (Figures 8C and 8D). Collectively, these results indicate that inhibition of CBP/p300 may produce an epigenetic conformation on the HIV-1 provirus that promotes transcriptional activation from the LTR, but that suppresses reversal of latency by HDAC inhibitors.

### Inhibition of PCAF/GCN5 or KAT6A/KAT6B reactivate latent HIV-1

**Figure 9:**
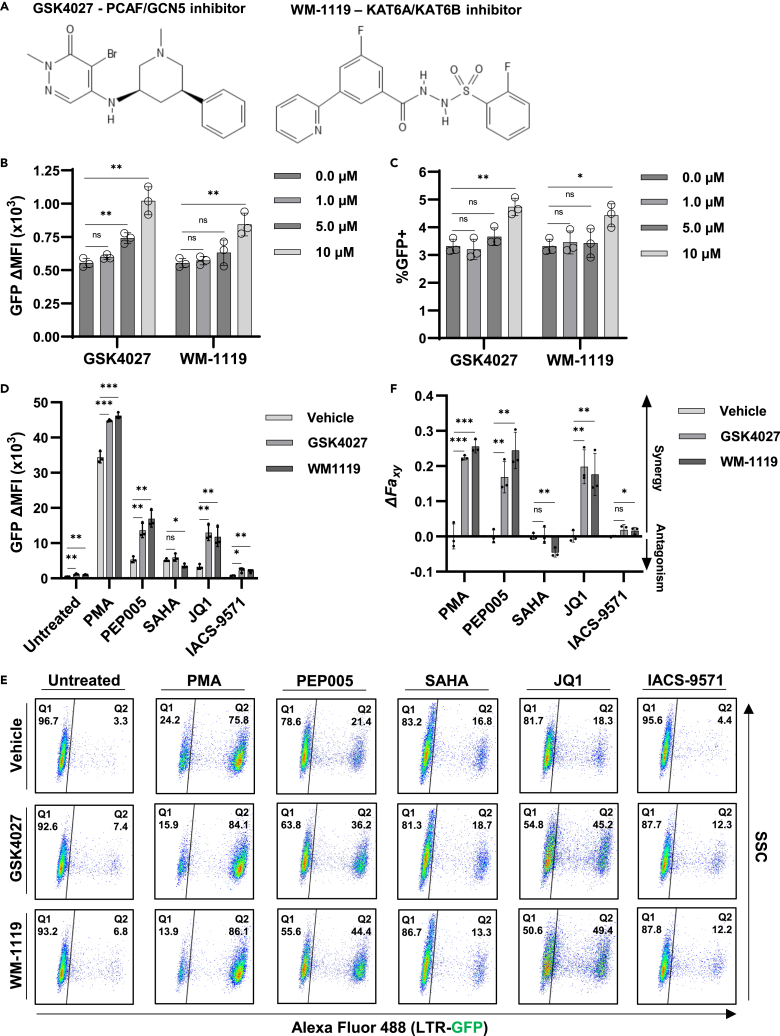
Inhibitors of various acetyltransferases induce proviral transcription (A) Chemical structure of GSK4027, a highly selective PCAF/GCN5 bromodomain inhibitor, and WM-1119, a highly selective KAT6A/KAT6B acetyltransferase inhibitor. (B and C) JLat10.6 cells were incubated with the indicated concentration of GSK4027 or WM-1119. Following 24 h, HIV-1 transcription was assessed by flow cytometry and is stated as the delta (Δ) GFP mean fluorescence intensity (MFI) (B) and the percentage of GFP positive cells (C) (*n* = 3, mean ± SD, unpaired t test). (D) JLat10.6 cells were pre-treated with a vehicle control (DMSO), 10 μM GSK4027, or 10 μM WM-1119 for 1 h prior to the addition of the indicated latency reversing agent. After 24 h, HIV-1 expression was assessed by flow cytometry and is reported as the GFP delta (Δ) mean fluorescence intensity (MFI). The concentration of LRAs used were 4 nM PMA, 4 nM PEP005, 1 μM SAHA, 10 μM JQ1, 15 μM IACS-9571 (*n* = 3, mean ± SD, unpaired t test). (E) Representative flow cytometry scatterplots for JLat10.6 cells treated as indicated in (D). The latent population is in Q1 (GFP−) while the transcriptionally active population is shown in Q2 (GFP+). (F) Bliss Independence Modeling was applied to determine combinatorial drug interaction. Values greater than 0 indicate synergy while values less than 0 signify an antagonistic relationship (*n* = 3, mean ± SD, unpaired t test). Statistical significance is indicated at ∗*p* < 0.05, ∗∗*p* < 0.01, or ∗∗∗*p* < 0.001, with n.s. denoting non-significant *p* ≥ 0.05.

The previous results indicate that CBP/p300 has a repressive effect for expression from the HIV-1 LTR. Additional lysine acetyltransferases are thought to be involved in productive HIV-1 infection, including PCAF and GCN5.^39^ To determine the contribution of these additional acetyltransferases for the regulation of HIV-1, we used GSK4027, a highly specific bromodomain inhibitor of PCAF/GCN5,^65^ and WM-1119, a specific KAT6A/KAT6B acetyltransferase inhibitor^66^ (Figure 9A). Treatment of JLat10.6 cells with either inhibitor caused a dose-dependent increase in expression of GFP from the LTR, although the PCAF/GCN5 inhibitor GSK4027 caused a greater effect than the KATA/KATB inhibitor WM-1119 (Figures 9B and 9C). Notably, reactivation by the PCAF/GCN5 and KAT6A/KAT6B inhibitors was markedly less than that caused by CBP/p300 inhibition (Figures 1B, 1C, and 3B). We also examined the effect of GSK4027 and WM-1119 in combination with various LRAs, where we found that either acetyltransferase caused enhanced viral expression with PMA, PEP005, JQ1, or IACS-9571, compared to either treatment alone (Figures 9D and 9E). Interestingly, PCAF/GCN5 inhibition had no effect on latency reversal by the HDACi SAHA, while KAT6A/KAT6B inhibition decreased reactivation by SAHA (Figures 9D and 9E, SAHA). Bliss Independence Modeling confirmed that the acetyltransferase inhibitors GSK4027 and WM-1119 caused synergistic effects for reactivation of HIV-1 in combination with PMA, PEP005, JQ1, and IACS-9571, but an antagonistic effect with SAHA (Figure 9F). Importantly, none of the treatments caused cellular toxicity in these experiments (Figure S6). Taken together, these results indicate that lysine acetyltransferases have both positive and negative regulatory functions regulation of HIV-1 expression in T cells.

## Discussion

cART has greatly improved the prognostic outcome of PLWH. However, cART does not represent a cure, and a cocktail of drugs must be administered for the entirety of the infected individual’s life.^1^ Given the persistence of HIV-1, recent research efforts have examined potential strategies toward eliminating antiretroviral treatment, the proposed “shock and kill” and “block and lock” strategies that would involve treatment with LRAs or LPAs, respectively.^4^ Clinical trials have already been performed using LRAs, but none have successfully caused reduction in the population of latently infected T cells.^67^ Also, various LPAs that suppress HIV-1 expression have been identified, but no agent with this capability has proceeded to clinical trials.^5^ Overall, implementation of these potential curative therapeutic strategies will require further characterization of mechanisms regulating HIV-1 latency.

Stochastic HIV-1 reactivation is thought to occur in part due to fluctuations in histone acetylation, causing chromatin opening.^68^ With the goal of producing epigenetically silenced HIV-1 provirus to induce deep latency, we targeted the acetyltransferase activity of CBP/p300 using the highly specific inhibitors A-485 and iP300w. To our surprise, small molecule inhibition of CBP/p300 acetyltransferase resulted in reversal of proviral latency *in vitro* (Figure 1) and in CD4^+^ cells from infected people *ex vivo* (Figure 4). To rule out the possibility that upregulation was caused by unexpected off target effects, we also used the PROTAC dCBP-1 to degrade CBP and p300 via the ubiquitin/proteasome pathway, where we observed effects that mirrored that of the acetyltransferase chemical inhibitors, resulting in a dose-dependent increase in proviral transcription (Figure 3). Furthermore, we found that CBP/p300 inhibitors produce synergistic effects with LRAs whose mechanism of activation of PKC, BRD4 bromodomain inhibition, or TRIM24 bromodomain inhibition, while antagonizing SAHA-mediated reactivation (Figures 2 and 3). Consistent with the finding that CBP/p300 inhibition limits latency reversal in response to HDAC inhibition, we found that CBP/p300 activity contributes to placement of H3K27ac and H3K4me3 at the LTR (Figures 7 and 8). Furthermore, small molecule inhibitors of the acetyltransferases PCAF/GCN5 and KAT6A/KAT6B were also shown to have latency reversal activity (Figure 9), although to a lesser extent than CBP/p300 inhibitors.

Acetylation of histone N-terminal tail lysine residues neutralizes the ε-amino group resulting in decompaction of adjacent nucleosomes and are epigenetic marks typically associated with transcriptional activation.^69^ A number of factors, including YY1, LSF, and NF-κB p50 homodimers bind to the LTR and recruit redundant HDACs leading to hypoacetylation of histones and suppression of HIV-1 transcription.^35^^,^^70^ Consistent with the role of HDACs for proviral silencing, HDACi’s have long been known to reverse latency by causing global histone acetylation. This understanding led to a focus on HDACi’s as LRAs for “shock and kill” clinical trials, with a number of trials performed over the past two decades.^37^ However, the results of these have not been encouraging as they failed to reduce the size of the latent viral reservoir despite causing a detectable increase in HIV-1 mRNA.^71^ The work presented here demonstrates that CBP/p300 in addition to other acetyltransferases including PCAF, GCN5, KAT6A, and KAT6B, repress HIV-1 transcription. This finding has important implications regarding the use of HDACi’s for “shock and kill” strategies as although HDAC inhibition succeeds in reactivating a distinct proviral population, the balance between protein acetylation and deacetylation is likely more nuanced for regulation of HIV-1 expression. Furthermore, while CBP/p300 activity causes repression of a subset of cellular genes is repressed, acetylation more frequently is associated with gene activation.^57^ Given the suppressive effect of CBP/p300 on HIV-1 transcription, this indicates that the retrovirus has evolved to produce a stochastic expression pattern that is divergent from the majority of cellular genes.

Mechanistically, we observe that CBP/p300 inhibition reduces LTR associated H3K27ac and H3K4me3 (Figures 7C and 7D). A large number of diverse proteins bind chromatin through reader domains including bromodomains, plant homeodomains, and YEATS domains.^72^ It is possible that CBP/p300 acetyltransferase activity creates an epigenetic context that either enables recruitment of transcriptional activators or displaces transcriptional repressors from the HIV-1 LTR. Recent studies have shown that acetylation of H4 causes recruitment of BRD4, which inhibits proviral transcription.^63^^,^^64^ However, the effect of CBP/p300 for reactivation of HIV-1 occurs independently from H4ac/BRD4, as chemical inhibition did not alter association of BRD4 with the LTR (Figure 7G). CBP/p300, PCAF/GCN5, and KAT6A/KAT6B are canonical HATs, which predominantly localize to the nucleus. However, they display differential acetylation of histone lysine residues; acetylation of H3K18 and H3K27 by CBP/p300 is well characterized, PCAF/GCN5 is known to target H3K9, and KAT6A/KAT6B show preference for H3K9, H3K14, and H3K23.^69^ As inhibitors of these acetyltransferases reverses latency, further characterization of LTR epigenetic composition will be necessary to establish whether the latency reversing effect is caused by histone modification.

In addition to histones, human cells express hundreds of non-histone nuclear proteins,^57^^,^^73^ and consequently HATs are often referred to as lysine acetyltransferases (KATs). HAT/KAT mediated protein acetylation is involved in cellular processes including gene transcription, cell cycle regulation, DNA damage repair, and autophagy.^73^ In particular, non-histone protein acetylation is thought to be a major regulator of transcription as acetylation is implicated in regulating the function of over 100 transcription factors, co-factors, and nuclear receptors.^73^ Interestingly, protein acetylation is independent of linear sequence motif suggesting that CBP/p300 produces an “acetyl-spray”, modifying accessible lysine residues on proximal proteins.^57^ This being the case, it seems probable that CBP/p300 represses HIV-1 through acetylation of non-histone proteins. In this scenario, CBP/p300 acetylation must inhibit a transcriptional activator and/or enhance function of a transcriptional repressor. Additionally, it is possible that latency reversal in response to acetyltransferase inhibition occurs by indirect factors such as increased availability of transcriptional activators. Our results demonstrate a novel effect of HAT/KAT function for regulation of HIV-1 transcription. Because HIV-1 transcription is regulated by numerous cellular factors that cause activation or repression from the LTR, and the function of most non-histone protein acetylation has not been defined, it may be difficult to identify specific lysine acetylation(s) that inhibit HIV-1 expression. Importantly, because specific protein acetylation may activate or repress HIV-1 provirus expression, successful implementation of chromatin modifying drugs for therapy will require a more detailed understanding of how protein acetylation controls function of the many factors that bind the HIV-1 LTR promoter.

### Limitations of the study

This research resulted in the unexpected observation that CBP/p300 acetyltransferase inhibitors cause reactivation of HIV-1 provirus, indicating that factors regulating expression of the latent provirus must be negatively regulated by lysine acetylation. Our results indicate this effect is likely caused by acetylation of non-histone protein targets. Identification of specific targets for p300/CBP that inhibit HIV-1 expression will be challenging because there are likely thousands of nuclear acetylated proteins. Identification of regulatory targets will be necessary in future studies to clarify the mechanism by which acetylase inhibitors cause reactivation of provirus expression, and establish whether the effect is imposed by direct control at the LTR or caused indirectly by a global alteration in protein function.

## Resource availability

### Lead contact

Further information and requests for resources and reagents should be directed to and will be fulflled by the lead contact, Ivan Sadowski (sadowski@mail.ubc.ca).

### Materials availability

This study did not generate any new material.

### Data and code availability

• RNA Seq. data are deposited to GEO and are publicly available. Accession number is also listed in the key resources table.
• This paper does not report original code.
• Any additional information required to reanalyze the data reported in this paper is available from the lead contact upon request.

## Acknowledgments

We thank LeAnn Howe and Zabrina Brumme for helpful comments for helpful comments. We also thank Z.B. and the BC Center for Excellence in HIV/AIDS for providing purified PBMCs from study participants. This research was supported by program project grant F16-01210, from the 10.13039/501100000024Canadian Institutes of Health Research, and F19-05392 Discovery Grant from the 10.13039/501100000038Natural Sciences and Engineering Research Council of Canada (to I.S.).

## Author contributions

R.M.H. and I.S conceived the experimental design. R.M.H. performed all experiments. R.M.H. and I.S. wrote the manuscript.

## Declaration of interests

The authors declare no conflicts of interest.

## STAR★Methods

### Key resources table

**Table:** 

REAGENT or RESOURCE	SOURCE	IDENTIFIER
**Antibodies**		

Tubulin (W.B.)	Abcam	Cat# ab7291;RRID: AB_2241126
p300 (W.B.)	Cell Signaling Technology	Cat# 54062; RRID: AB_2799450
CBP (W.B.)	Abcam	Cat# ab253202
Goat Anti-Rabbit-HRP (W.B.)	Abcam	Cat# ab6721; RRID: AB_955447
Goat Anti-Mouse-HRP (W.B.)	Pierce	Cat# 1858413
CD16/CD32 Rat anti-Mouse, Clone 2.4G2 (FLOW)	BD Biosciences	Cat# 553141; RRID: AB_394656
PE-Cy™7 Mouse Anti-Human CD69 Clone FN50 (FLOW)	BD Biosciences	Cat# 561928; RRID: AB_10895389
PE-Cy™7 Mouse Anti-Human CD25 Clone M-A251 (FLOW)	BD Biosciences	Cat# 561405; RRID: AB_10646034
Rabbit IgG (ChIP)	Abcam	Cat# ab1722730
H3K27ac (ChIP)	Abcam	Cat# ab4729; RRID: AB_2118291
H3K4me3 (ChIP)	Abcam	Cat# ab213224; RRID: AB_2923013
H3K9ac (ChIP)	Abcam	Cat# ab32129; RRID: AB_732920
H4K5ac (ChIP)	Abcam	Cat# ab51997; RRID: AB_2264109
BRD4 (ChIP)	Abcam	Cat# ab314432
RNAPII (ChIP)	Abcam	Cat# ab26721; RRID: AB_777726
RNAPII S2P (ChIP)	Abcam	Cat# ab238146

**Bacterial and virus strains**		

Red-Green-HIV-1	Dahabieh et al.^22^	N/A

**Biological samples**		

Human Peripheral Blood CD4^+^ T-cells	StemCell Technologies	200–0165

**Chemicals, peptides, and recombinant proteins**		

A-485 CBP/p300 inhibitor	Tocris	6387
iP300w CBP/p300 inhibitor	Tocris	7270
dCBP-1	MedChemExpress	HY-134582
Phorbol 12-myristate 13-acetate (PMA)	MilliporeSigma	P1585
PEP005	Tocris	4054
Suberoylanilide hydroxamic acid (SAHA)	MilliporeSigma	SML0061
JQ1	Tocris	4499
IACS-9571	MedChemExpress	HY-102000B

**Critical commercial assays**		

Power SYBR® Green RNA-to-CT™ 1-Step Kit (RT-PCR)	Thermo Fisher	4402955

**Deposited data**		

Jurkat E6-1 RNA-Seq. T cell activation	Horvath^18^	GEO: GSE227850

**Experimental models: Cell lines**		

Jurkat E6-1	–	–
JLat10.6	–	–
JLatA72	–	–

**Oligonucleotides**–		

RT-PCR Forward for HIV-1 multiply spliced Tat-Rev: CTTAGGCATCTCCTATGGCAGGA	This study	N/A
RT-PCR Reverse for HIV-1 multiply spliced Tat-Rev: GGATCTGTCTCTGTCTCTCTCTCCACC	This study	N/A
RT-PCR *GAPDH* Forward: TGCACCACCAACTGCTTAGC	This study	N/A
RT-PCR *GAPDH* Reverse: GGCATGGACTGTGGTCATGAG	This study	N/A
RT-PCR *IL2* Forward: AACTCACCAGGATGCTCACA	This study	N/A
RT-PCR *IL2* Reverse: GCACTTCCTCCAGAGGTTTGA	This study	N/A
RT-PCR *CD69* Forward: TCTTTGCATCCGGAGAGTGGA	This study	N/A
RT-PCR *CD69* Reverse: ATTACAGCACACAGGACAGGA	This study	N/A
RT-PCR *IL8* Forward: ACTGAGAGTGATTGAGAGTGGAC	This study	N/A
RT-PCR *IL8* Reverse: AACCCTCTGCACCCAGTTTTC	This study	N/A
RT-PCR *CSF2* Forward: ACCTGCCTACAGACCCGCCT	This study	N/A
RT-PCR *CSF2* Reverse: GAAGTTTCCGGGGTTGGAGGGC	This study	N/A
RT-PCR *CCL22* Forward: CGCGTGGTGAAACACTTCTAC	This study	N/A
RT-PCR *CCL22* Reverse: GCCACGGTCATCAGAGTAGG	This study	N/A
RT-PCR *XCL1* Forward: CTCCTTGGCATCTGCTCTCT	This study	N/A
RT-PCR *XCL1* Reverse: GCTCACACAGGTCCTCTTATC	This study	N/A
ChIP-qPCR E-Box Forward: GTGAGCCTGCATGGAATGGA	This study	N/A
ChIP-qPCR E-Box Reverse: CGGATGCAGCTCTCGGG	This study	N/A
ChIP-qPCR RBE3 Forward: AGCCGCCTAGCATTTCATC	This study	N/A
ChIP-qPCR RBE3 Reverse: CAGCGGAAAGTCCCTTGTAG	This study	N/A
ChIP-qPCR NF-κB Forward: TTTCCGCTGGGGACTTTC	This study	N/A
ChIP-qPCR NF-κB Reverse: CCAGTACAGGCAAAAAGCAG	This study	N/A
ChIP-qPCR RBE1 Forward: AGTGGCGAGCCCTCAGAT	This study	N/A
ChIP-qPCR RBE1 Reverse: AGAGCTCCCAGGCTCAAATC	This study	N/A
ChIP-qPCR Gag Forward: AGCAGCCATGCAAATGTTA	This study	N/A
ChIP-qPCR Gag Reverse: AGAGAACCAAGGGGAAGTGA	This study	N/A

### Experimental model and study participant details

Samples from HIV-1 infected participants were collected with written informed consent under a protocol jointly approved by the research ethics boards at Providence Health Care/UBC and Simon Fraser University (certificate H16-02474). We were not able to establish influence of sex or gender on the results of this study because of the limited size of the participant population.

All cell lines were routinely tested for mycoplasma. Jurkat E6-1, JLat10.6, JLatA72, cell lines were cultured in Roswell Park Memorial Institute 1640 Medium (RPMI-1640) supplemented with 10% Fetal Bovine Serum (FBS), penicillin [100 units/ml], streptomycin [100 g/mL], and L-glutamine [2 mM]. HEK293T cells were cultured in Dulbecco’s Modified Eagle’s Medium (DMEM) supplemented with 10% FBS, penicillin [100 units/ml], streptomycin [100 g/mL], and L-glutamine [2 mM]. All tissue cultures were incubated in a humidified 37°C and 5% CO_2_ atmosphere. Peripheral Blood Mononuclear Cells (PBMCs) from participants with HIV-1 on ART were isolated from whole blood by density gradient centrifugation using Lymphoprep and SepMate tubes (StemCell Technologies), and cryopreserved. Human Peripheral Blood CD4^+^ T-cells, purchased from StemCell Technologies (Catalog #200-0165, Lot #2107423002). Upon thawing, PBMCs were cultured in RPMI supplemented with 10% FBS, penicillin [100 units/ml], streptomycin [100 g/mL], L-glutamine [2 mM], and 40 U/mL IL2.

### Method details

#### Cell culture and viral infection

Vesicular stomatitis virus G (VSV-G) pseudotyped viral stocks were produced by co-transfecting HEK293T cells with a combination of viral molecular clone, psPAX, and VSV-G at a ratio of 8 μg: 4 μg: 2 μg. Transfections were performed with polyethylenimine (PEI) at a ratio of 6 : 1 (PEI: DNA) in Gibco Opti-MEM. Lentiviral infections were performed by plating 1x10^6^ cells in 24-well plates with 500 μL RPMI containing 8 μg/mL polybrene and the amount of viral supernatant to give the desired multiplicity of infection (MOI). Plates were spinoculated for 1.5 h at 1500 rpm. For RGH infection, T-cells were incubated with Dynabeads Human T-Activator CD3/CD28 beads for three days. Subsequently, the beads were removed, 8 μg/mL polybrene and RGH virus was added, and cells were spinoculated for 2 h at 1500 rpm and 32^o^C. Cells were stained with Gibco Trypan Blue Solution (0.4%) and viability was determined using a Bio-Rad TC20 Automated Cell Counter.

#### Analysis of protein, mRNA and reporter gene expression

Cells treated as indicated in the legends were lysed in RIPA buffer (50 mM Tris-HCl pH 7.5, 150 mM NaCl, 1% Triton X-100, 1 mM EDTA, 0.1% SDS, 0.5% sodium deoxycholate, 2.5 mM PMSF) at 4^o^C. Equivalent amounts of cellular extracts were mixed with 5× SDS–PAGE sample buffer, boiled for 3 min, separated on SDS–PAGE gels, and transferred to nitrocellulose membrane. The membrane was blocked with 2% milk (w/v) in TBS with 0.1% Tween for 1 h at room temperature and then incubated with a primary antibody overnight at 4°C. Subsequently, the membrane was incubated for 1 h at room temperature with a secondary HRP antibody in 2% milk (w/v) in TBS with 0.1% Tween after which the autoradiograph was developed. Antibodies used are as follows: Tubulin, Abcam ab7291, 1:13333; p300, Cell Signaling Technology #54062, 1:1000; CBP, Abcam ab253202, 1:2000; Goat Anti-Rabbit-HRP, Abcam ab6721, 1:1100000; Goat Anti-Mouse-HRP, Pierce 1858413, 1:20000. Full-length western blots are presented in Figure S7.

RNA was extracted from cells using the RNeasy Kit (Qiagen) and analyzed with the Quant Studio 3 Real-Time PCR system (Applied Biosystems) using *Power* SYBR Green RNA-to-CT 1-Step Kit (Thermo Fisher) as per the manufacturer’s instructions. RT-PCR data was normalized to GAPDH expression using the ΔΔCt method as previously described.^18^ Cycling parameters were as follows: [48°C, 30 min], 1x; [95°C, 10 min], 1x; [95°C, 15 s, 60°C, 1 min], 50x.

For flow cytometry, cells were treated as indicated in the legends and suspended in PBS. Flow cytometry was performed using a BD Biosciences LSRII-561 system with threshold forward scatter (FSC) and side scatter (SSC) parameters set so that a homogeneous population of live cells was assessed. GFP delta (Δ) Mean Fluorescence Intensity (MFI) specifies that the MFI of the Jurkat E6-1 negative control sample has been subtracted. For surface staining of membrane receptors, 1x10^6^ Jurkat E6-1 cells were treated as indicated. Following treatment, cells were washed 1x with PBS on ice and resuspended with 500 μL FACS Buffer (1x PBS, 2% FBS, 5 mM EDTA). Blocking was then performed by adding CD16/CD32 Rat anti-Mouse, Clone 2.4G2 (BD Biosciences 553141) and incubating on ice for 15 min with rotation. Subsequently, 0.8 μg of PE-Cy7 Mouse Anti-Human CD69 Clone FN50 (BD Biosciences 561928) or 0.8 μg of PE-Cy7 Mouse Anti-Human CD25 Clone M-A251 (BD Biosciences 561405) was added. Samples were incubated for 30 min on ice with rotation and then washed 2x with FACS Buffer and analyzed by flow cytometry.

#### ChIP-qPCR and RNA-Seq. Analysis

JLat10.6 cells were fixed with 1% formaldehyde (Sigma-Aldrich) for 10 min at room temperature (1.5x10^7^ cells per ChIP). Crosslinking was quenched by the addition of 125 mM glycine for 5 min, after which cells were washed with PBS at 4^o^C. Cells were lysed in NP-40 Lysis Buffer (0.5% NP-40, 10 mM Tris-HCl pH = 7.8, 3 mM MgCl_2_, 1x PIC, 2.5 mM PMSF) for 10 min on ice. Following sedimentation, nuclei were resuspended in Sonication Buffer (10 mM Tris-HCl pH = 7.8, 10 mM EDTA, 0.5% SDS, 1x PIC, 2.5 mM PMSF) and sonicated using a Covaris S220 Focused-ultrasonicator to produce sheared DNA that predominately ranged from 200 to 2000 bp using the following settings: Treatment 1 [ Peak Power: 200, Duty Factor: 10, Cycles/Burst 200, Duration 30 s]; Treatment 2 [ Peak Power: 2.5, Duty Factor: 0.1, Cycles/Burst 50, Duration: 30 s]; Repeat 6x. The soluble chromatin fraction was collected and snap frozen in liquid nitrogen. Chromatin concentrations were normalized among samples and pre-cleared with Protein G agarose beads (Millipore, 100 μL). The chromatin samples were split in two and diluted with IP buffer (10 mM Tris-HCl pH = 8.0, 1.0% Triton X-100, 0.1% deoxycholate, 0.1% SDS, 90 mM NaCl, 2 mM EDTA, 1x PIC); samples were immunoprecipitated with the indicated specific antibody in parallel with a no antibody mock IP. Antibodies used for ChIP were: Rabbit IgG, Abcam ab1722730, 10 μg; H3K27ac, Abcam ab4729, 4 μg; H3K4me3, Abcam ab213224, 3 μg; H3K9ac, Abcam ab32129, 5 μg; H4K5ac, Abcam ab51997, 8 μg; BRD4, Abcam ab314432, 10 μg; RNAPII, Abcam ab26721, 4 μg; RNAPII S2P, Abcam ab238146, 5 μg.

The chromatin/antibody mixtures were incubated 1 h at 4^o^C with rotation. Pre-washed Protein G agarose beads (40 μL) were then added, and the samples were incubated overnight at 4^o^C with rotation. Bead/antibody complexes were washed 3x in Low Salt Wash Buffer (20 mM Tris-HCl pH = 8.0, 0.1% SDS, 1.0% Triton X-100, 2 mM EDTA, 150 mM NaCl, 1x PIC) and 1x with High Salt Wash Buffer (same as Low Salt but with 500 mM NaCl). Elution and crosslink reversal was performed by incubating 4 h at 65^o^C in elution buffer (100 mM NaHCO_3_, 1% SDS) supplemented with RNase A. DNA was purified using the QIAQuick PCR purification kit (QIAGEN) and ChIP DNA was analyzed using the Quant Studio 3 Real-Time PCR system (Applied Biosystems). The percent input value of the sample paired no antibody mock immunoprecipitation has been subtracted from the percent input obtained from immunoprecipitation with IgG or the indicated specific antibody of the corresponding sample. RNA-seq as analyzed by DESeq2 to identify genes differentially expressed upon T cell activation was obtained from NCBI GEO accession GSE227850.^18^ Volcano plots were generated using the Galaxy web platform^74^ while heatmaps depicting gene expression were created in RStudio.

### Quantification and statistical analysis

All replicates are presented as mean values with ±standard deviation shown by error bars. *P*-values were determined using GraphPad Prism 10.2.1 with the number of replicates and the statistical method used noted in the figure legends. Statistical significance is indicated at ∗*p* < 0.05, ∗∗*p* < 0.01, or ∗∗∗*p* < 0.001, with n.s. denoting non-significant *p* ≥ 0.05. FlowJo software (TreeStar) was used to analyze data and determine the indicated Mean Fluorescence Intensity (MFI) for flow cytometry experiments. Quantitative analysis of drug interactions was as previously described^75^ using Bliss independence modeling. The Bliss independence model is defined by the equation *Fa*_xy, P_ = *Fa*_*x*_ + *Fa*_*y*_ – (*Fa*_*x*_)(*Fa*_*y*_), whereby *Fa*_xy, P_ is the predicted fraction affected by a combination of drug *x* and drug *y* that is derived from the experimentally observed fraction affected by drug *x* (*Fa*_*x*_) and drug *y* (*Fa*_*y*_) individually. Comparison of the predicted combinatorial affect (*Fa*_xy, P_) with the experimentally observed impact (*Fa*_*xy*,O_) is then performed: Δ*Fa*_*xy*_ = *Fa*_*xy*,O_ − *Fa*_*xy*,P_. If Δ*Fa*_*xy*_ is greater than 0, the combination of drugs *x* and *y* exceed that of the predicted affect indicating that the drugs possess synergistic interaction. If Δ*Fa*_*xy*_ = 0, the drug combination follows the Bliss model for independent action. If Δ*Fa*_*xy*_ is less than 0, the drug interaction is antagonistic as the observed effect of the drug combination is less than predicted combined affect. In this analysis, the fraction affected was calculated as follows: *Fa*_*x*_ = (GFP ΔMFI of drug *x* – GFP ΔMFI untreated)/(maximum GFP ΔMFI obtained by any given treatment within the experiment – GFP ΔMFI untreated).
