# Supplementary material for: CBP/p300 lysine acetyltransferases inhibit HIV-1 expression in latently infected T cells
Source: iScience. 2024 Oct 28;27(12):111244. doi: 10.1016/j.isci.2024.111244 (PMC11617383; doi:10.1016/j.isci.2024.111244)
Supplement: Document S1. Figures S1–S7 [file mmc1.pdf]

iScience, Volume *27*

Supplemental information

CBP/p300 lysine acetyltransferases inhibit HIV-1 expression in latently infected T cells

Riley M. Horvath and Ivan Sadowski

Figure S1

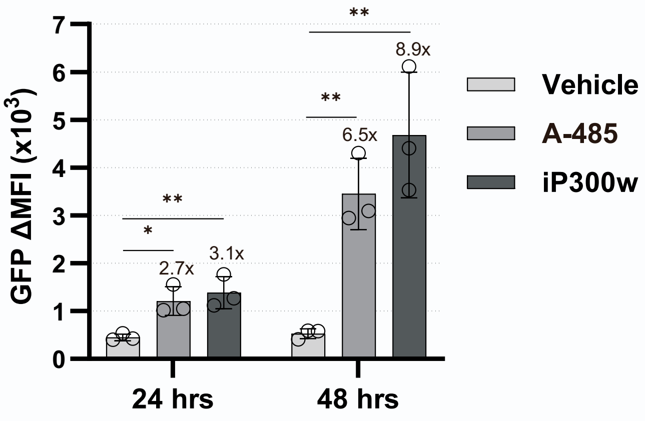
GFP ΔMFI (x10³)
0
1
2
3
4
5
6
7
*
**
**
**
2.7x
3.1x
6.5x
8.9x
24 hrs
48 hrs
Vehicle
A-485
iP300w

Figure S2

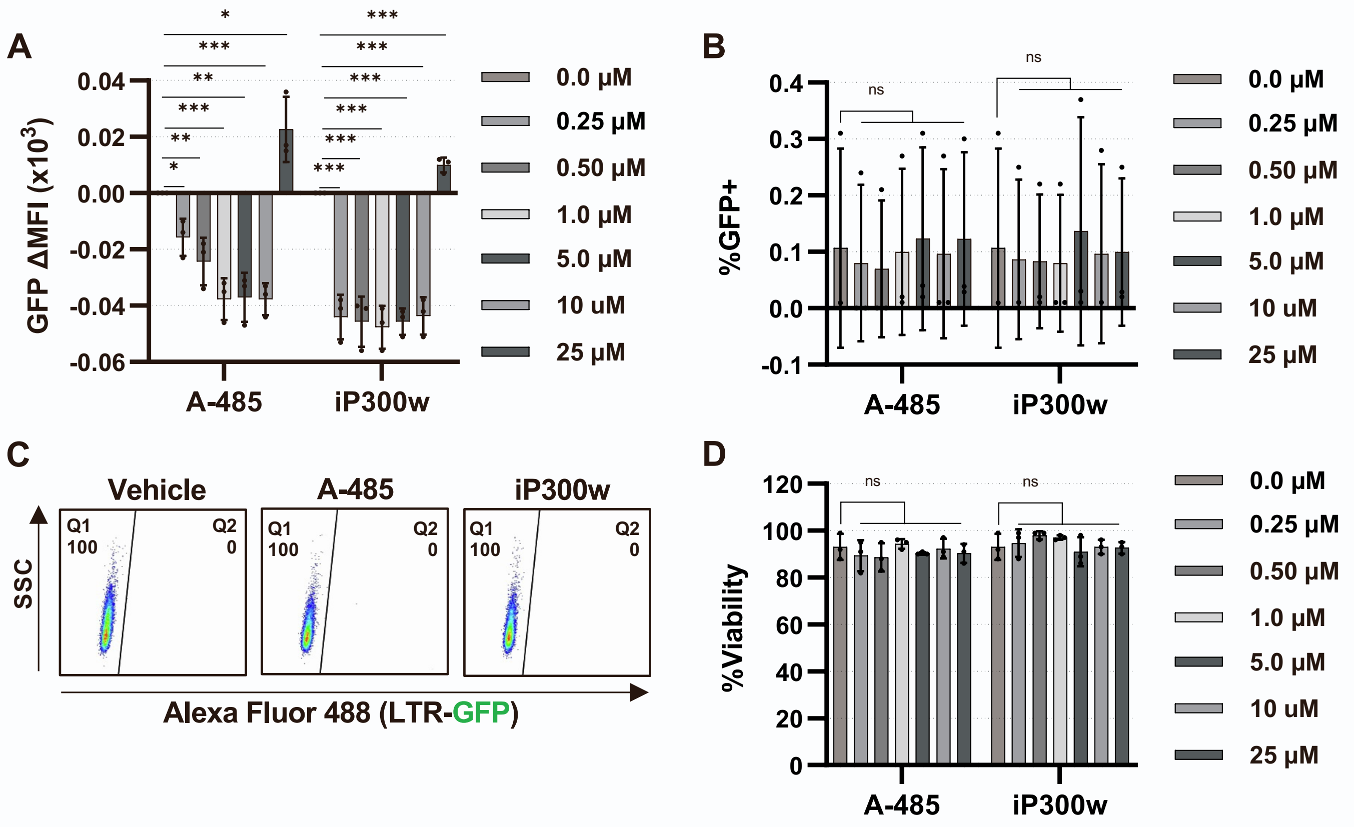
A
GFP ΔMFI (x10³)
A-485
iP300w
0.0 μM
0.25 μM
0.50 μM
1.0 μM
5.0 μM
10 uM
25 μM
B
%GFP+
ns
A-485
iP300w
C
Vehicle
A-485
iP300w
SSC
Q1 100
Q2 0
Alexa Fluor 488 (LTR-GFP)
D
%Viability
ns
A-485
iP300w

Figure S3

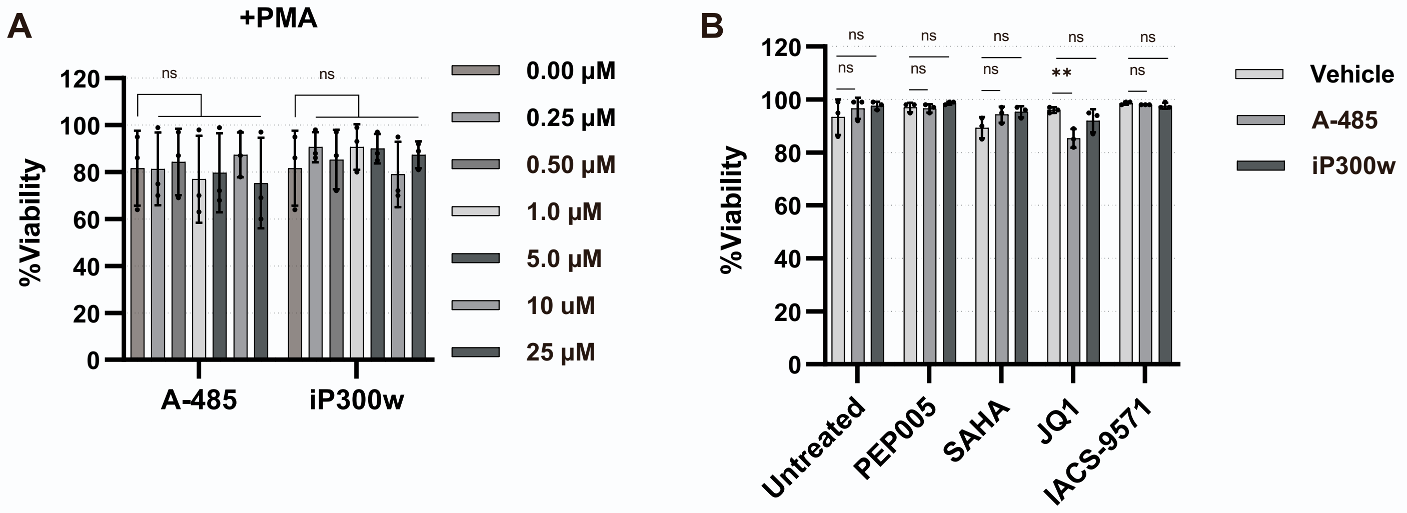
A
+PMA
ns
ns
120
100
80
60
40
20
0
%Viability
A-485
iP300w
0.00 µM
0.25 µM
0.50 µM
1.0 µM
5.0 µM
10 uM
25 µM
B
ns
ns
ns
ns
ns
ns
ns
ns
**
ns
120
100
80
60
40
20
0
%Viability
Untreated
PEP005
SAHA
JQ1
IACS-9571
Vehicle
A-485
iP300w

Figure S4

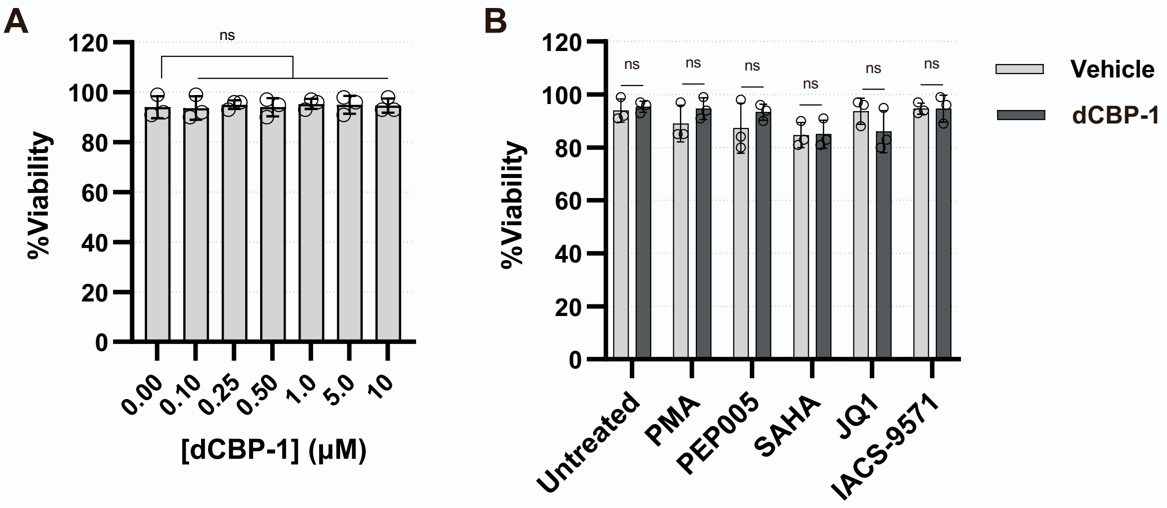
A
ns
120
100
80
60
40
20
0
%Viability
0.00
0.10
0.25
0.50
1.0
5.0
10
[dCBP-1] (μM)
B
ns
120
100
80
60
40
20
0
%Viability
Untreated
PMA
PEP005
SAHA
JQ1
IACS-9571
Vehicle
dCBP-1

Figure S5

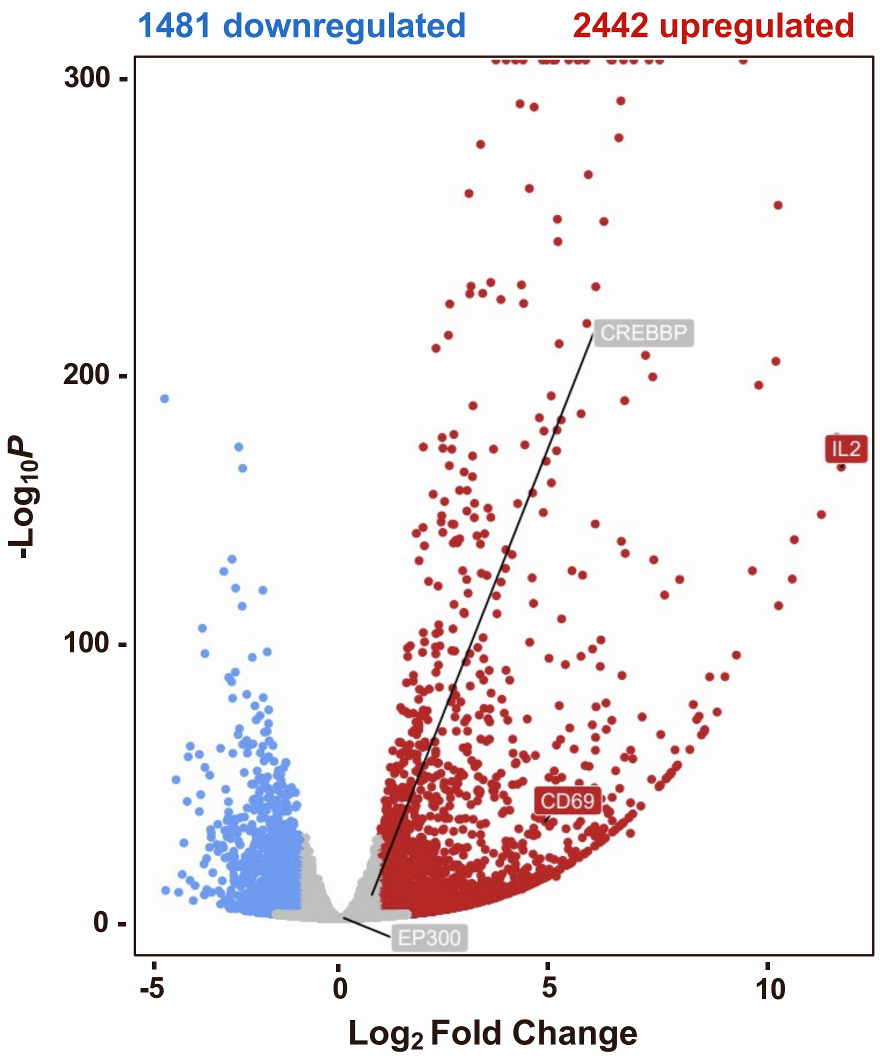

1481 downregulated
2442 upregulated
300
200
100
0
-Log10P
-5
0
5
10
Log2 Fold Change
CREBBP
IL2
CD69
EP300

Figure S6

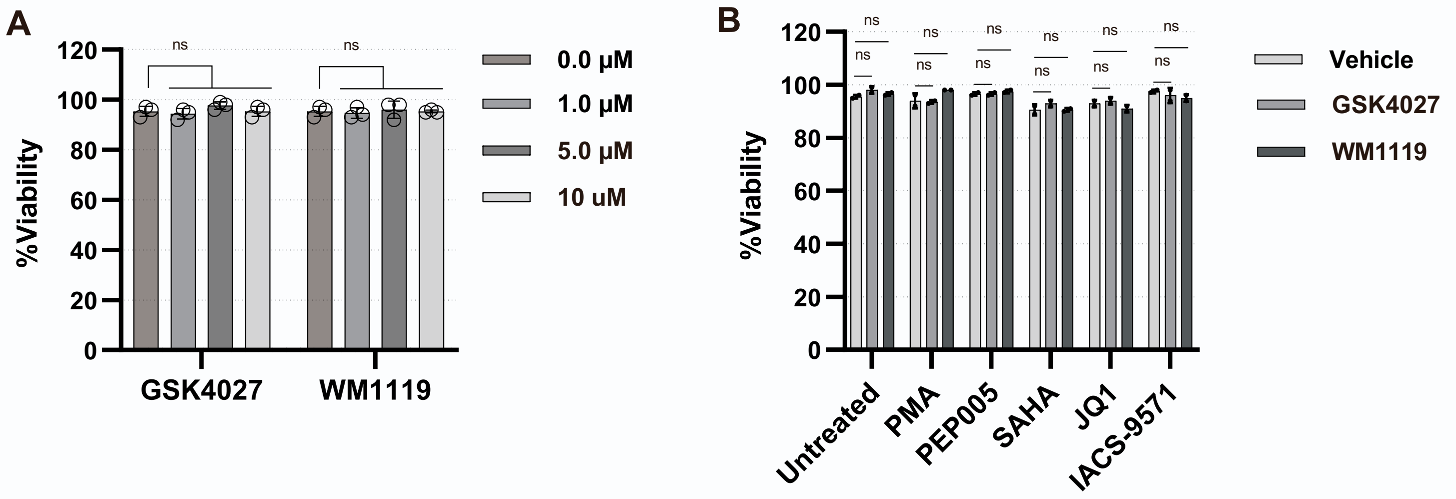
A
%Viability
ns
0.0 μM
1.0 μM
5.0 μM
10 uM
GSK4027
WM1119
B
%Viability
ns
Vehicle
GSK4027
WM1119
Untreated
PMA
PEP005
SAHA
JQ1
IACS-9571

Figure S7

Figure 3*A*

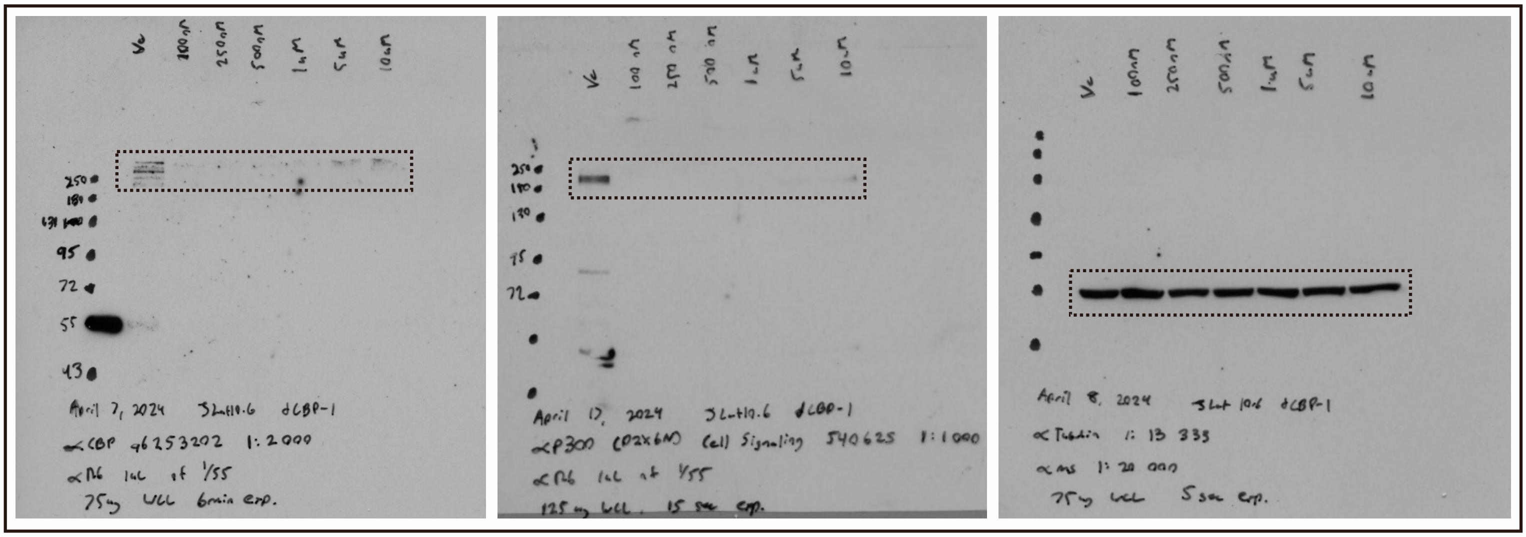

Legends to Supplementary Figures

Figure S1. CBP/p300 inhibition causes greater proviral reactivation over time. JLat10.6 cells were incubated with a vehicle control (DMSO), 10 μM A-485, or 10 μM iP300w. Following incubation for 24 hrs or 48 hrs, proviral expression was determined by flow cytometry and is indicated as the GFP delta (Δ) Mean Fluorescence Intensity (MFI) ($n = 3$, mean ± SD, unpaired *t*-test).

Figure S2. A-485 and iP300w do not cause autofluorescence. A, B: Jurkat E6-1 cells were incubated with the indicated concentration of A-485 or iP300w. Subsequent to 24 hrs, flow cytometry was performed to assess GFP as measured by delta (Δ) Mean Fluorescence Intensity (MFI) (A) and the percentage of GFP positive cells (B) ($n = 3$, mean ± SD, unpaired *t*-test). **C:** Representative flow cytometry scatter plots of Jurkat E6-1 cells treated as in (A, B). **D:** Viability was determined for Jurkat E6-1 cells treated as in (A, B) ($n = 3$, mean ± SD, unpaired *t*-test).

Figure S3. Cellular viability following treatment with CBP/p300 inhibitors in combination with LRAs. A: Following a 1 hr pre-treatment with the indicated concentration of A-485 or iP300w, 4 nM PMA was added to JLat10.6 cells. Cellular viability was determined after 24 hr incubation ($n = 3$, mean ± SD, unpaired *t*-test). **B:** JLat10.6 cells were pre-treated with DMSO (Vehicle), 10 μM A-485, or 10 μM iP300w for 1 hr after which the indicated LRA was added. The concentration of LRA used was 4 nM PEP005, 1 μM SAHA, 10 μM JQ1, and 15 μM IACS-9571. Viability was subsequently determined ($n = 3$, mean ± SD, unpaired *t*-test).

Figure S4. Effect of dCBP-1 on cellular viability. A: JLat10.6 cells were incubated with the indicated concentration of dCBP-1 for 24 hrs after which cellular viability was assessed (n = 3, mean ± SD, unpaired t-test). **B:** Following a 1 hr pre-treatment with 1 μM dCBP-1, the indicated LRA was added, and cellular viability was determined after 24 hrs. The concentration of LRA used was 4 nM PMA, 4 nM PEP005, 1 μM SAHA, 10 μM JQ1, and 15 μM IACS-9571 (n = 3, mean ± SD, unpaired t-test).

Figure S5. RNA-seq of activated T-cells. Volcano plot depiction of genes that are differentially expressed following the treatment of Jurkat T-cells with PMA/ionomycin as analyzed by DESeq2 [1].

Figure S6. Effect of GSK4027 or WM-1119 on cellular viability. A: JLat10.6 cells were incubated in the presence of the indicated concentration of GSK4027 or WM-1119 for 24 hrs after which cellular viability was determined (n = 3, mean ± SD, unpaired t-test). **B:** Following a 1 hr pre-treatment with a Vehicle control (DMSO), 10 μM GSK4027, or 10 μM WM-1119, the indicated LRA was added and after 24 hrs, cellular viability determined. The concentration of LRA used was 4 nM PMA, 4 nM PEP005, 1 μM SAHA, 10 μM JQ1, and 15 μM IACS-9571 (n = 3, mean ± SD, unpaired t-test).

Figure S7. Full-length western blots.

Original unaltered immunoblot exposures for Figure 3.

References

1. Horvath, R.M., Dahabieh, M., Malcolm, T., and Sadowski, I. (2023). TRIM24 controls induction of latent HIV-1 by stimulating transcriptional elongation. Commun Biol *6*, 86. https://doi.org/10.1038/s42003-023-04484-z.
